# A New Model and Method for Understanding *Wolbachia*-Induced Cytoplasmic Incompatibility

**DOI:** 10.1371/journal.pone.0019757

**Published:** 2011-05-10

**Authors:** Benjamin Bossan, Arnulf Koehncke, Peter Hammerstein

**Affiliations:** Institute for Theoretical Biology, Humboldt University, Berlin, Germany; University of California Merced, United States of America

## Abstract

*Wolbachia* are intracellular bacteria transmitted almost exclusively vertically through eggs. In response to this mode of transmission, *Wolbachia* strategically manipulate their insect hosts' reproduction. In the most common manipulation type, cytoplasmic incompatibility, infected males can only mate with infected females, but infected females can mate with all males. The mechanism of cytoplasmic incompatibility is unknown; theoretical and empirical findings need to converge to broaden our understanding of this phenomenon. For this purpose, two prominent models have been proposed: the mistiming-model and the lock-key-model. The former states that *Wolbachia* manipulate sperm of infected males to induce a fatal delay of the male pronucleus during the first embryonic division, but that the bacteria can compensate the delay by slowing down mitosis in fertilized eggs. The latter states that *Wolbachia* deposit damaging “locks” on sperm DNA of infected males, but can also provide matching “keys” in infected eggs to undo the damage. The lock-key-model, however, needs to assume a large number of locks and keys to explain all existing incompatibility patterns. The mistiming-model requires fewer assumptions but has been contradicted by empirical results. We therefore expand the mistiming-model by one quantitative dimension to create the new, so-called goalkeeper-model. Using a method based on formal logic, we show that both lock-key- and goalkeeper-model are consistent with existing data. Compared to the lock-key-model, however, the goalkeeper-model assumes only two factors and provides an idea of the evolutionary emergence of cytoplasmic incompatibility. Available cytological evidence suggests that the hypothesized second factor of the goalkeeper-model may indeed exist. Finally, we suggest empirical tests that would allow to distinguish between the models. Generalizing our results might prove interesting for the study of the mechanism and evolution of other host-parasite interactions.

## Introduction

*Wolbachia* are a group of 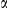-proteobacteria that infect a major proportion of insect species (see [1], [2] for reviews). They are known for intricate manipulations of their host's reproduction. The most puzzling manipulation is called Cytoplasmic Incompatibility (CI). In males, CI consists of *Wolbachia* manipulating the sperm in a yet unknown way – this manipulation is called mod (for modification). DNA from modified sperm cannot properly participate in the first embryonic mitosis, except if *Wolbachia* action in the egg recovers the functionality of the sperm DNA. This recovery is called resc (for rescue) and without it, embryos derived from modified sperm often exhibit high mortality rates [3], [4].

Owing to the nature of CI, a female can only successfully mate with an infected male if she is herself infected by an appropriate *Wolbachia* strain. If such an infected female mates with an uninfected male, there are no defects. Therefore, infected females have a selective advantage over uninfected ones, helping *Wolbachia* spread. Considering that CI effectively inhibits certain crosses, *Wolbachia* infection could lead to reproductive isolation or gene flow reduction between host populations with different infection statuses [5]–[7]. Therefore, CI *Wolbachia* may play an important role in insect speciation [8]–[10]. A deeper insight into the mechanism behind *Wolbachia-*induced CI is thus likely to further our understanding of host evolutionary dynamics.

How *Wolbachia* accomplish to induce cytoplasmic incompatibility is still unclear. One promising attempt to explain this phenomenon is the mistiming-model. It states that CI *Wolbachia* induce a desynchronization in cellular events. After fertilization, sperm modification leads to delayed progression of the male pronucleus. Similarly, ovum manipulation leads to delayed progression of the female pronucleus [11] or, more likely, of cell cycle timing [12]. If only sperm is modified, the increased time needed for the male pronucleus to participate in mitosis could exceed the time available and incompatibility may occur. If neither sperm nor ovum are modified or if both are modified by the same degree, synchrony is restored and the embryo develops as usual. Moreover, defects do not occur if only the ovum is modified, as the slowed cell cycle inhibits the male pronucleus from beginning mitosis preemptively. Two important aspects distinguish the mistiming-model from other models. First it is based on the experimental finding that the male pronucleus lags behind in incompatible crosses of *Nasonia vitripennis* and *Drosophila simulans* [4], [11], [13]. Second, the same type of manipulation would be sufficient to induce mod in sperm and resc in ova.

Poinsot *et al.* [14] systematically examined whether several models can explain the facts known about *Wolbachia*-induced CI. They presented six CI patterns derived from laboratory experiments and assessed their consistency with the different candidate models. The authors found that the mistiming-model cannot account for some observations. For example, the fact that some CI-inducing *Wolbachia* strains cannot rescue one another (bidirectional incompatibility) cannot be explained; in the mistiming-model, given two strains, either the first should rescue the second or the other way round. The authors also attempted to alter the mistiming-model so that it can account for all findings but, as we will show, these attempts cause new problems.

**Figure 1:**
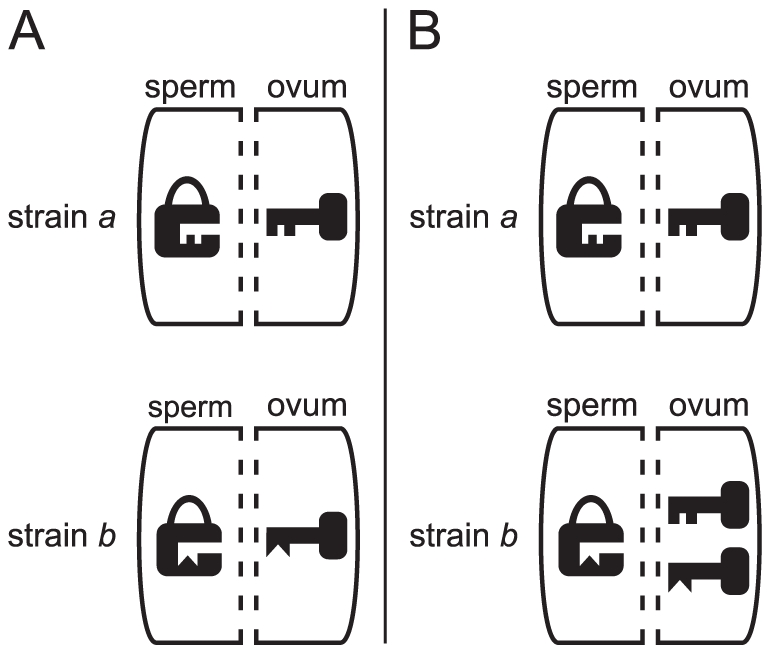
How bidirectionally and unidirectionally incompatible *Wolbachia* strains are represented in the lock-key-model. (A) strains 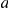 and 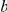 are bidirectionally incompatible: Neither 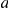 nor 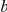 has the key to each other's lock. (B) 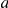 and 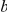 are unidirectionally incompatible: 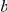 has the key to 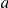
's lock, but 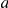 does not have the key to 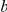
's lock.

According to the analysis by Poinsot *et al.* [14], the best account for the facts is given by the lock-key-model. In this model, *Wolbachia* deposit “locks” to the paternal DNA that render these chromosomes unable to participate in mitosis, whereas in the egg cytoplasm, *Wolbachia* deposit the matching “keys” that recover the functionality of the paternal DNA (Fig. 1). If all locks are matched by corresponding keys, a mating is compatible. By assuming that different strains produce different pairs of locks and keys, bidirectional incompatibility can be explained. The lock-key-model also explains the other known CI patterns. However, molecular evidence for the existence of locks and keys is lacking [14].

The major conceptual differences between mistiming and lock-key is that only in the first model, mod and resc are the same function, and that the first model is quantitative, whereas the second is qualitative. The mistiming-model focuses on the length of delay, which is supposed to vary among *Wolbachia* strains. The lock-key-model, on the other hand, does not distinguish between the quantity of every lock and key but only considers whether every type of lock is matched by the corresponding key. Assuming that locks and keys are different molecular mechanisms implies that they are encoded by different genes; this assumption is not required for the mistiming-model.

Since the mistiming-model fails to account for all empirical findings, we propose to add another quantitative dimension besides timing and call the resulting model goalkeeper-model. Imagine a soccer goalkeeper who has to catch a penalty shot. For that to happen, she must jump far enough and high enough (ignore that she could jump too high or too far). Jumping far enough but not high enough will lead to a goal, as would jumping high enough but not far enough. Similarly, for CI not to occur, the goalkeeper-model requires two conditions to be met. The first condition could be that the time available for the male pronucleus to prepare must exceed the time needed, as in the mistiming-model. The second condition could, for example, be related to prophage activity [15]. We discuss the evidence for the existence of a second condition for CI in a later section.

Our goal in this work is to evaluate lock-key and goalkeeper with a new method discussed in the Methods section. In short, we translate the models' verbal descriptions into a set of logical rules. These rules allow us to deduce whether certain statements are true within the formal framework of the models. We conceptualized the critical variables of the models as “factors”, so that within the models' frameworks, mistiming and goalkeeper are based on factor magnitude, whereas lock-key is based on factor type.

Our approach will allow us to address the question whether the models can explain the known CI patterns. A minimal requirement for the models to be valid is that they are compatible with the stylized facts A-F from Poinsot *et al.* [14] (see Table 1). Furthermore, we will subject the models to additional tests that allow for other observations on CI: (1) whether the models can explain the diverse compatibility relationships observed among *Wolbachia* strains; (2) whether it is possible to derive predictions with regard to CI levels, and if so, whether these predictions are correct; (3) how well the models are supported by cytological evidence; and finally how model choice is influenced by evolutionary considerations regarding (4) double infections or novel compatibility types and (5) the origin of CI. We will also propose tests that allow to distinguish between the two models.

**Table 1:** The most interesting statements and their truth values according the goalkeeper-model and the lock-key-model.

	Statement	goalkeeper	lock-key
A	If only in the ovum but not in the sperm, *Wolbachia* does not cause CI	true	true
B	Bidirectional incompatibility is possible	true	true
C	Unidirectional incompatibility is possible	true	true
D	Additional strains in males cannot decrease mod strength	true	true
D′	Even if strain 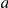 rescues strain 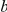 , it cannot rescue the double-infection 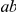 (except if 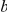 is [mod−])	true	false
D″	If strain 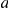 rescues strain 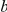 , it also rescues the double-infection 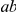	false	true
E	Additional strains in females cannot decrease resc strength	true	true
E′	The double-infection 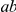 rescues the mono-infection 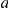	true	true
F	The existence of [mod− resc+] strains is possible	true	true
H	There are strains 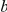 and 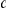 that cannot rescue 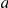 by themselves but can do so together	true	true
I	Intransitivity: It is possible that 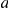 rescues 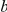 which rescues 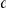 , but still 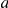 cannot rescue 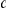	true	true
J	If 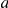 rescues the double-infection 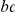 , then the double infection 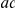 rescues the triple-infection 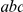	false	true
K	Only if 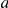 rescues 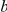 does the double-infection 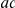 rescue the double-infection 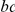	true	false
M	There are strains that are [mod−] in one host and [mod+] in another	true	not derivable
P	If 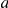 rescues 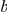 , and if 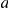 rescues 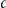 , it also rescues the double-infection 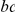	false	true

Evidence: A: [28, and many others], B: [28], [37], C: [16], [17], [19], [38], D: [16], [23], [38], [39], E: [16], [22], [23], [38], E′: [16], [22], F: [35], [40], I: [17], [19], M: [19], [35]. Formal proofs: Text S1.

Statements A-F are from [14]. It is assumed that each strain can rescue itself.

## Results and Discussion

### Comparison of CI patterns predicted by the models

**Figure 2:**
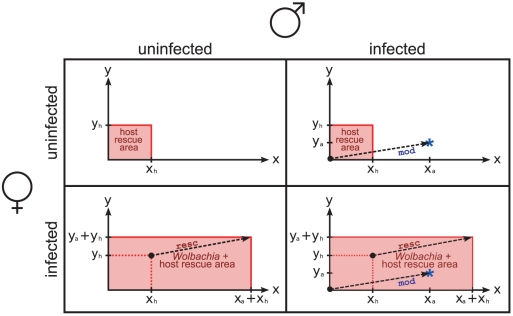
How the goalkeeper model's two quantitative factors produce the known CI patterns. Females and males can either be uninfected or infected by *Wolbachia*. Two factors, 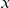 and 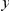, are involved in the generation of CI. *Wolbachia* contribute 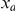 to factor 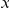 and 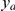 to factor 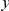 in equal amounts during modification in males and rescue in females (dashed arrows). Hosts contribute the net host contribution 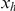 to factor 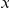 and 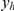 to factor 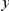 in females only. Rescue occurs within the red areas, either due to hosts only (top row) or in combination with *Wolbachia* (bottom row). The blue asterisk shows the modification by *Wolbachia* (right column). CI occurs only if this blue asterisk does not lie within the rescue area because this implies that at least one of the factors 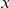 or 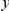 is produced at greater quantity in males than in females (top right).

We subjected the goalkeeper-model and the lock-key-model to an analysis using formal logic that allows to derive unambiguous properties of the models. While we leave the formal proofs to Text S1, we give a quick intuition for how the goalkeeper-model explains the basic features of cytoplasmic incompatibility (CI). From the point of view of the goalkeeper model, two factors 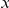 and 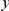 are involved in the differential generation of CI in crosses between and among infected and uninfected mating partners (see Fig. 2). The quantities of these two factors are specific to each *Wolbachia* strain. In infected males, the factors contribute to modification of sperm, and in infected females, they contribute to rescue in ova. Hosts also support rescue by adding the host specific ‘net host contribution’ 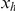 and 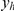. This assumption reflects a robustness requirement that the host would have to meet–perhaps to a lower degree–even in the absence of *Wolbachia*. In order to assess whether CI occurs after fertilization, the amounts of resc and mod factors are compared. CI manifests if and only if at least one mod factor exceeds the corresponding resc factor in quantity. Note that a specific *Wolbachia* strain 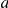 has the same effect in females and males–if the female is infected, strain specific amounts 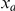 and 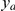 are contributed to rescue; if the male is infected, the same amounts 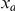 and 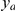 are contributed to modification.

The formal analysis revealed that both the goalkeeper-model and the lock-key-model are not contradicted by known facts (see Table 1). Therefore, they are both promising contenders. Nevertheless, the two models sometimes make differential predictions. For example, according to statement D′ (Table 1), even if a *Wolbachia* strain 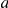 rescues strain 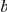, it cannot rescue the double-infection by 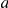 and 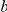. The only exception is if strain 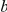 is [mod−], that is, if it does not cause CI anyway. This statement is true in the goalkeeper-model and false in the lock-key-model. On the other hand, according to D″, if strain 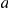 rescues strain 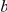, it is always able to rescue the double-infection by 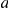 and 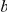. This statement is false in the goalkeeper-model and true in the lock-key-model.

Differential predictions such as D′ and D″ suggest potential experiments that can confirm one model and falsify the other. One possible experiment would be to (1) confirm that both the *Wolbachia* strains wMel and wRi induce CI; (2) confirm that the double-infection of wMel and wRi induces CI; (3) confirm that wRi rescues wMel; (4) test whether a double-infection of wMel and wRi can be rescued by wRi. If the assumptions are confirmed and if wRi rescues the double-infection, the goalkeeper-model is falsified. If, however, wRi does not rescue the double-infection, the lock-key-model is falsified. Other tests could be done that would allow to reject (at least) one of the models, but this particular experiment is easy to perform, can be realized with the currently available equipment, and is unambiguous.

We also analyzed the validity of the mistiming-model. Confirming the results of Poinsot *et al.* [14], our tests showed that the mistiming-model cannot support some of the facts, for example the existence of bidirectional incompatibility. To make the mistiming-model consistent with bidirectional incompatibility, we incorporated the changes proposed by Poinsot *et al.* [14], namely that different factors with different binding sites might exist (details on this analysis can be found in Text S1). While these changes allow the mistiming-model to account for bidirectional incompatibility, they lead to contradictions with other empirical findings. Resolving these contradictions requires incorporating further factors into the model, making it less parsimonious than the goalkeeper-model. That is why we believe that the goalkeeper-model, which includes mistiming as a special case, is the more promising approach to explaining CI.

### Compatibility relationships

**Figure 3:**
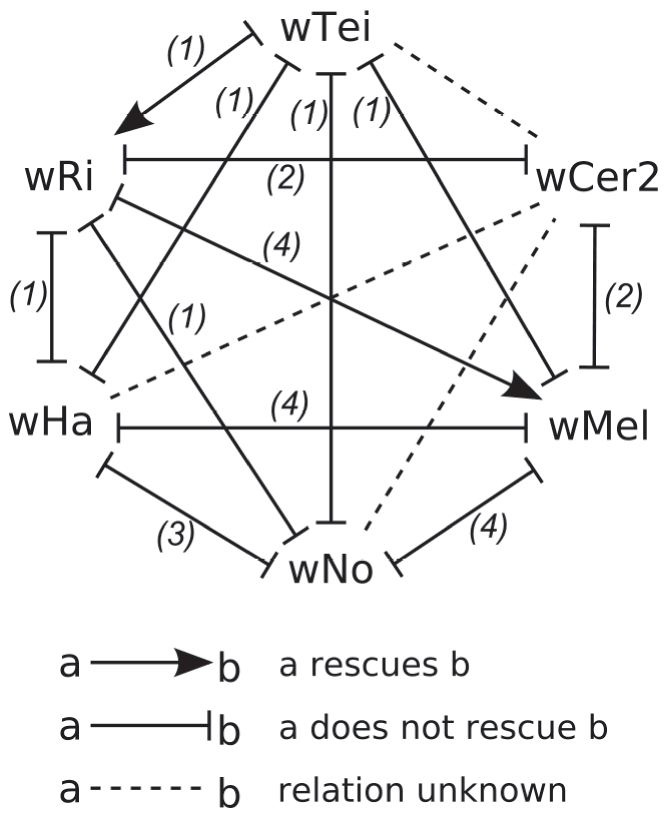
The compatibility relationships of six studied *Wolbachia* strains. These relationships were used to study whether the goalkeeper-model and the lock-key-model are able to reproduce empirical data. Threshold for CI: corrected CI level of 20%. The host species is *D. simulans* in all studies. References: (1) [19], (2) [18], (3) [16], (4) [17].

We used data gathered in previous studies [16]–[19] to determine the compatibility relationship of six *Wolbachia* strains. As far as the data showed, wCer2, wNo, and wHa are bidirectionally incompatible with all other strains, whereas wTei rescues wRi but not *vice versa*, wRi rescues wMel but not *vice versa*, and wMel is bidirectionally incompatible with wTei. The resulting compatibility relationships are represented in Fig. 3. The goal was then to test whether the two models can reproduce this compatibility relationship.

**Figure 4:**
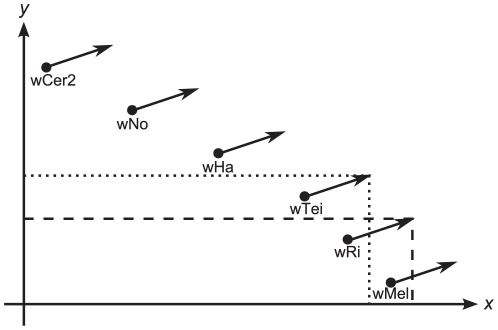
Explanation of the compatiblity relationship using the goalkeeper-model. The points represent the contribution by the corresponding *Wolbachia* strain to the two mod factors (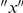 and 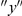). The black arrow represents the host contribution to rescue. The contribution of a strain to the two resc factors equals its own contribution plus the net host contribution. When both resc factors exceed both mod factors in quantity, rescue is successful. Thus wTei with the help of the net host contribution rescues wRi (indicated by wRi being within the dotted frame). In contrast, wRi can rescue wMel but not wTei (indicated by wMel but not wTei being within the dashed frame).

Our analysis shows that the goalkeeper-model can be fitted to many different compatibility relationships despite the model's mere two factors. In the example of the six *Wolbachia* strains we studied, a possible distribution of the two factors that can explain the data is presented in Fig. 4. This compatibility relationship would not be possible without assuming that the host contributes to the amount of rescue factors (black arrow). To summarize, we could show that the goalkeeper-model can explain the experimentally found compatibility relationships among these six *Wolbachia* strains.

In the lock-key-model, for a strain to be bidirectionally incompatible with another strain, some of its locks must be unmatched by the keys of the other strain and *vice versa*. Correct distribution of locks and keys can also account for unidirectional incompatibility. However complex the compatibility relationships among strains, the lock-key-model could generate that pattern by assuming a sufficient number of different locks and keys. More interesting than generating a specific compatibility relationship is thus to determine how many locks and keys must be involved *at least* to explain the relationships. For the data from the six *Wolbachia* strains we studied, a minimum of five factors has to be assumed. We illustrate a possible distribution of locks and keys in Table 2. Interesting to note, the number of involved mod and resc factors can be smaller than the number of involved strains, as has previously been shown by [20]. Zabalou *et al.* [19] stated that their findings cannot be explained except if wTei possesses at least three resc factors; this statement is substantiated by our analysis of the lock-key-model. However, a quantitative model like the goalkeeper-model can account equally well for such complex compatibility relationships as studied here while using only two factors.

**Table 2:** Explanation of the compatibility relationship using the lock-key-model.

	wTei		wRi		wHa		wNo		wMel		wCer2	
factor	mod	resc	mod	resc	mod	resc	mod	resc	mod	resc	mod	resc
1	L	K			L	K	L	K			L	K
2	L	K	L	K							?	
3		K	L	K					L	K	?	
4				K	L	K			L	K	?	
5							L	K			?	

Presence of locks is indicated by “L”, presence of keys “K”, unknown relationships by question mark. A strain rescues another strain if all its keys match the other's locks.

### CI levels

A CI model should preferably not only be able to predict in which crosses CI occurs but also what CI level to expect. Although we did not directly model CI levels, some reasonable predictions can be derived from the goalkeeper-model. These predictions are especially apparent when hosts are infected by multiple CI-inducing *Wolbachia* strains. They are: 1) more *Wolbachia* strains in females should decrease the CI level, because they increase the amount of resc factors; 2) more *Wolbachia* strains in multiply infected males should increase the CI level, because they increase the amount of mod factors; 3) equivalent crosses should lead to similar CI levels, because the difference in the amount of mod and resc factors should be equal. These predictions rely on the assumption that the CI level is proportional to the norm of the difference of the vectors representing mod and resc factors, as shown in Fig. 2. Note, however, that predictions of type 1) and 2) assume that in multiple infections, the density of the *Wolbachia* strains is not reduced compared to the respective densities of the single infections, an assumption which does not always hold [21].

To test the three predictions, we used data published in previous works [16], [22], [23]. As the data often did not allow to reverse calculate significance levels, we simply tested whether the predictions pointed to the right direction – whether CI levels indeed decreased (1), increased (2), or were within a 10% margin (3). Of 60 predictions that could be made, 45 were qualitatively correct and 14 false (1 draw). This result is highly significant in favor of the taken approach (p<0.005, 1-tailed binomial test); predictions of type 1 were the most accurate. For lack of original data, though, this tentative analysis should be treated with care. Corroborating our findings, ANOVA tests showed that in *Leptopilina heterotoma*, CI levels induced by three *Wolbachia* strains always differed significantly except if the crosses were equivalent [24]. Moreover, in multiple infections in the flower bug *Orius strigicollis*, eight out of ten equivalent crosses did not produce statistically significant differences in CI levels [21], which supports predictions of type 3. The fact that none of the findings of [21] produced significant results in favor of the predictions of type 2, may be due to a reduced *Wolbachia* density in multiple infections. However, such density-reductions were not found in other studies [25], [26]. Therefore, the goalkeeper-model's most straightforward extension assumes that density is not reduced during multiple infections. Future models could include the possibility of density reduction.

For lock-key, it is difficult to expand such a strictly qualitative CI model to account for differences in CI levels. One approach is to assume that CI levels depend on the number of different keys matching the locks. Charlat *et al.* [18], [27] studied a lock-key-model with ten possible locks and keys. They assumed that if for a total of ten locks, six keys match, the CI level would be 40%. Conclusive tests on whether such a model can explain the data on CI levels have not been performed yet.

### Cytological evidence

The two hypothetical factors of the goalkeeper-model should correspond to events during embryonic or larval development. The model could imply that one of the factors (say 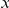) is a time delay, as mistiming does. Then the quantity this factor contributes to mod corresponds to the increase of the time needed to prepare the male pronucleus for mitosis, and the quantity this factor contributes to resc corresponds to the increase of the time available to prepare the male pronucleus for mitosis. If the time available is less than the time needed (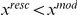), the male pronucleus would not be ready when mitosis starts, resulting in segregational defects. If the time available is greater than or equal to the time needed (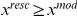), the male pronucleus could participate in mitosis, no matter how large the difference. The host contribution could be interpreted as a tolerance time that the female pronucleus or the zygote as a whole can wait before it is too late for the male pronucleus to participate. This way mistiming could be included in the goalkeeper-model.

If the 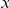-factor is responsible for defects in cell cycle timing during the first mitosis, the other factor (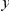) probably involves events at a later stage of development. CI-induced defects during later stages have been observed indirectly. For example, it was found that although 76% of the *Drosophila* embryos derived from incompatible crosses died owing to CI, only 56% showed defects during the first cell cycle, leaving 20% of embryonic deaths unexplained [3]. This finding strongly suggests that CI affects one fifth of the embryos at a later developmental stage. Given the lack of observable defects during the first cell cycle, these deaths are unlikely to be simple after-effects. It was observed that some *Wolbachia* strains in *Culex pipiens* caused host mortality during later developmental stages [15]. These findings support the hypothesis that one of the two factors conjectured by the goalkeeper-model is indeed the mistiming of cell cycle events, whereas the other factor is unrelated to these defects. As a counterexample, it was found that in incompatible crosses of *Drosophila simulans*, the percentage of embryos displaying defects during the first cell cycle corresponded exactly to the CI level [28]. However, the reason for this observation could be that the first factor was the sole determinant of incompatibility in these crosses (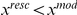 and 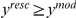). Moreover, because this factor is probably the first to matter, we would expect defects during the first mitotic division to be the most frequent, albeit not sole cause of CI.

The exact biological mechanism behind the second factor cannot be derived from the logical framework of the goalkeeper-model. Although we do not want to speculate too intensely, there are some hints that the *Wolbachia* prophage WO is involved in causing CI. For example, a link has been found between CI patterns and a prophage protein in *C. pipiens* [29], [30]. Conveniently, this host species also exhibits CI-caused embryonic mortality occurring after the first cell cycle. Thus it is possible that the second factor is related to the *Wolbachia* prophage. The finding that in some host species there is no positive correlation between prophage and CI [31] or that some CI inducing *Wolbachia* strains do not possess the prophage [32] are not contradictory to this idea. It is possible that the CI produced by the corresponding *Wolbachia* strains is only caused by the first factor, whereas the other strains operate with both factors. If this is the case, one would expect *Wolbachia* strains of the first group to only exhibit unidirectional but not bidirectional incompatibility with each other, a proposition that can be tested. In summary, it is likely that CI involves more than just defects during the first cell cycle, and this finding could be accounted for by a conjectured second factor, which might be related to the WO prophage.

The lock-key-model has different implications as to how CI is induced cytologically. The most frequently proposed interpretation of the lock-key-model is that mod consists of different factors binding to the paternal DNA, and resc consists of other factors that remove the mod factors [14]. All mod factors need to be removed to stop CI from occurring. Unfortunately, there is no cytological evidence to date that hints at the existence of such lock and key type of gene products [14]. Quite in contrast, closely related *Wolbachia*-strains with different compatibility relationships but no apparent genetic differences have been found [33]. The reason could be that the genetic differences were simply not detected, but this fact can be accounted for more easily by the goalkeeper-model, according to which the difference in mod and resc capabilities does not lie in different gene products but in different gene expression levels.

### Population genetics

The predictions derived from goalkeeper-model and the lock-key-model can also be tested by evolutionary reasoning. For example, statement D″ (Table 1) can be extended to make conclusions on real insect populations. According to D″, if a *Wolbachia* strain 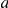 can unidirectionally rescue strain 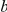, it can also rescue the double-infection 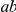. Therefore, the mono-infection by 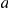 could rescue each cross that the double-infection 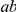 can rescue. Still the double-infection has the disadvantage that, without synergistic interactions, its transmission rate must necessarily be less than that of the mono-infection. The double-infection, being strictly inferior to the mono-infection, should not be able to persist in natural populations. Hence we would expect never to find an insect population containing a double-infection of unidirectionally incompatible strains. However, D″ is only true for the lock-key-model but not for the goalkeeper-model. The latter predicts instead that if the male is double-infected, only a double-infected female can successfully mate with it (statement D′ in Table 1). Thus, in contrast to the lock-key-model, the goalkeeper-model allows double-infections of unidirectionally incompatible strains to persist. These contrasting findings demonstrate that the CI mechanism can have important implications for the evolutionary dynamics of double infections, which in turn can be used to test the models.

We can also address the question whether a mutant *Wolbachia* strain that is bidirectionally incompatible with the wildtype strain can evolve within an infected population. This problem has been studied by Charlat *et al.* [34] who assumed that mod and resc function can evolve independently from each other. This separate evolution is possible in the lock-key-model but not in the goalkeeper-model, in which per assumption mod and resc are the same function. These authors have shown that the evolution of a new, bidirectionally incompatible strain is possible through emergence of a mutant *Wolbachia* strain that cannot rescue its own modification but rescues the modification of the wildtype *Wolbachia* strain. Assuming that there is no sib mating, this “suicidal” mutant can spread through random drift. At this point, a second mutant could spread if it can rescue the “suicidal” strain; this second mutant would be bidirectionally incompatible with the initial wildtype strain. These findings lead to the suggestion (hypothesis G in [14]) that the evolution of new, bidirectionally incompatible *Wolbachia* strains is more likely if mod and resc can evolve independently.

In the goalkeeper-model, two strains are bidirectionally incompatible if one of them produces more of one factor but less of the other. As a result, mutants that are bidirectionally incompatible with the wildtype cannot spread easily. A new, bidirectionally incompatible *Wolbachia* strain vanishes because of its low initial frequency, and a new strain could only spread if it rescues the wildtype strain (unidirectional incompatibility). Therefore, bidirectionally incompatible mutants cannot spread under these “sympatric” circumstances. However, “allopatric” evolution of bidirectionally incompatible strains may still occur. If an infected host population divides into two, a *Wolbachia* mutant could appear in the first population that produces more of the first factor. Because it can unidirectionally rescue the wildtype strain, this mutant would spread and become fixed. In the second population, the same could happen, but instead more of the second factor is produced. Consequently, the first strain produces more of the first factor and the second strain more of the second factor. Thus, in the two populations, there would be two new *Wolbachia* strains that are bidirectionally incompatible. In summary, the goalkeeper-model and also the lock-key-model allow allopatric evolution of new, bidirectionally incompatible *Wolbachia* strains, but only the latter allows sympatric evolution of such strains.

### Evolutionary origin of CI

Understanding the evolutionary origin of known CI patterns presents a challenge to the lock-key framework. We have shown that at least five types of locks and keys are required to explain the compatibility relationship of six studied *Wolbachia* strains. In *Culex pipiens*, a minimum of eight factors have to be assumed to explain the known compatibility relationships [20], [33]. More factors probably have to be assumed to explain the compatibility relationship of other *Wolbachia* strains.

The mod-resc system of CI can be interpreted as a poison-antidote system where mod corresponds to the poison and resc to the antidote. Explaining how that many poisons and antidotes evolved is difficult for lock-key because the model assumes that poison and antidote are different functions. As different functions are encoded by different genes and simultaneous emergence of both functions is unlikely, first one function must have evolved and then the other. But the existence of just one function does not convey a selective advantage for a *Wolbachia* strain. A neutral trait that is rare is expected to vanish quickly from a population, so a *Wolbachia* strain with this new property should be on a short clock to develop the other function. Moreover, as locks and keys need to match one another, it would not suffice to evolve some random lock or key, but the second factor must specifically fit the first. An escape route would be to assume that pleiotropic effects conveying a fitness advantage to bearers of this trait help to maintain the otherwise neutral mutation but to the authors' knowledge, no such effects have been observed yet.

In contrast, the goalkeeper-model needs not assume that mod and resc are different functions. Once mod is present, the same function would guarantee rescue if the female is infected. Moreover, the problem that in order to explain the compatibility types, very specific and thus rare mutations have to occur repeatedly, does not manifest in the goalkeeper-model. The goalkeeper-model is thus better suited to explain the origin of the known CI patterns.

As an additional advantage, the origin of the two factors of the goalkeeper-model can be deduced plausibly from the perspective of *Wolbachia*'s evolutionary past. Assuming quantitative factors, it is likely that the first embodiment of CI only involved one dimension, like pure mistiming. As a consequence, only unidirectional but not bidirectional incompatibility would have existed [14]. Therefore, strains that incorporate a second factor in their CI mechanism would be favored by selection relative to one-factor strains, as the latter could never rescue the first but *vice versa*. As we have seen, the existence of a second factor allows a countless number of bidirectionally incompatible *Wolbachia* strains to exist; this number does not increase if yet another factor is introduced. Given this and assuming that changing the gene expression level (different quantity) is easier than evolving a new functional protein (different quality), the selective pressure is low to acquire a third factor instead of just changing the quantity of the existing ones. Thus a stop at two factors seems to be a plausible evolutionary outcome for a quantitative model.

The logic of lock-key implies that some *Wolbachia* strains possess excess keys; for instance, unidirectional incompatibility suggests this implication and intransitivity (fact I) absolutely requires it. The excess keys would not serve to rescue the very strain producing them but would only serve to rescue other strains. However, one strain randomly developing a key to another strain's locks is unlikely because of the great diversity of locks and keys. This unlikelihood is exacerbated if these strains never meet in nature, so that no selective pressure exists to develop these keys. However, it has been found that unrelated *Wolbachia* strains can rescue each other [33], [35]. Certainly, one could assume that the corresponding keys were acquired through common ancestry, but then one needs to answer why the functionality of these useless keys has not been lost in the evolutionary history.

By contrast, within the framework of the goalkeeper-model, the poison is simultaneously the antidote, and a *Wolbachia* strain only produces the rescue factors it requires to rescue itself. Since there are only two factors, one would expect that some strains produce enough of the two factors to rescue other strains. Therefore, the goalkeeper-model immediately suggests why sometimes unidirectional compatibility should occur.

To sum up, explaining the high number of locks and keys implied by the compatibility relationships between *Wolbachia* strains, as well as the existence of useless keys, is a difficult, though surely not insurmountable challenge for the lock-key-model. On the other hand, the goalkeeper-model can explain the origin of CI without invoking pleiotropic effects and, as we have seen, can explain all currently known patterns of CI occurrence. It thus constitutes a promising novel candidate mechanism for the generation of CI.

## Methods

### Comparison of CI patterns predicted by the models and compatibility relationships

Instead of using verbal analyses [14] to study whether the models can account for CI patterns, we use a framework based on formal logic. That is, each statement is derived logically from the models' particular sets of rules and not merely from verbal reasoning. This approach has four advantages: Underlying model assumptions are clarified; exact and unambiguous conclusions on whether a model can account for the data are possible; novel predictions can be formulated *ad libitum*; and different models can be compared with regard to their parsimony. A detailed account of our method is given in Text S1.

The assumptions made for the goalkeeper-model are the following. There are two factors that can be present in different positive quantities. When a host is infected by two or more different *Wolbachia* strains, the magnitude of each factor is assumed to be the added quantity of the corresponding factor contributed by each single strain. The change in quantity does not depend on the sex of the infected host. In addition, for the goalkeeper-model to account for certain findings (for example intransitivity, statement I in Table 1), we need to assume a certain contribution of female hosts to the two rescue factors. This additional assumption also makes the goalkeeper-model a useful framework to understand the influence of host genetic background on CI (some *Wolbachia* strains can be “[mod+]” and “[mod−]” depending on host background [36]; and see statements M, N, and O in Text S1). The overall aid from the female host is called net host contribution. A cross results in CI if at least one of the factors 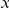 or 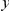 is produced at greater quantity in males than in females. The mistiming-model is identical to the goalkeeper-model, except that only one factor is implemented instead of two.

The lock-key-model works differently. The number of involved factors is not constrained. Also, a *Wolbachia* strain might produce more keys than are required to match its own locks. CI occurs if and only if one or several locks deposited in the sperm are not matched by a corresponding key deposited in the ovum. If a host is infected by more than one *Wolbachia* strain, it is assumed that the union of the locks or keys is produced. Net host contributions need not be assumed.

None of the two models is strictly more parsimonious than the other: Goalkeeper is more parsimonious with regard to only assuming two factors and mod and resc to be the same function; lock-key is more parsimonious with regard to being independent of factor quantity and not needing to assume net host contributions.

One could expand lock-key by making it quantitative so that the amount of individual matching locks and keys would matter [18], [27]. However, then suddenly only two factors would need to be assumed and one would not need to assume that mod and resc are different functions. In other words, the most parsimonious form of a quantitative lock-key-model could be reduced with few adaptations to the presented goalkeeper-model. Therefore, and given that lock-key, as is, can already account for all known data, we refrained from making it quantitative.

To test the models' explanatory power concerning the compatibility relationships found between different *Wolbachia* strains, we used several sources of empirical data [16]–[19]. CI levels were extracted from these works to calculate the corrected CI levels [17], measured as the percentage of embryonic mortality. The limit of what CI level counts as incompatible was set at 20%. We removed from consideration all *Wolbachia* strains that were incompatible with none or only one of the other strains.

The discovery of the “suicide strain” wTei which cannot rescue its own modification [19] presents a problem for the studied models. The goalkeeper-model implies that each strain should be able to rescue itself. The lock-key-model could be changed so as to allow a strain to produce more locks than keys. wTei could then produce one or several locks but not the corresponding keys so as to be suicidal. This would mean, however, that wTei should not be able to rescue itself in its natural host, *Drosophila teissieri*. This is not the case [35]. Therefore, the lock-key-model would be falsified by these findings as well. Therefore, either both models must be false or the data must present some flaws.

A closer look at all the compatible crosses performed by Zabalou *et al.* [19] reveals that (averaged over the results from both laboratories) wTei causes a CI level of 41.5%, wRi of 34.9% and wMel of 39.0%. As these mortality rates are exceptionally high, wRi and wMel should be called suicidal, too. Yet these strains are known for a long time and have never been characterized as suicidal (see e.g. [16], [18], [22]). Therefore, it seems that in the work by Zabalou *et al.* [19], some of the CI levels derived from compatible crosses (that is, crosses in which males and females are infected by the same set of strains) are unusually high. We can but speculate about the causes of this anomaly. For example, the combination of introgression and cytoplasmic injection used by the authors to produce the infected *Drosophila* strains could have resulted in unusually low growth rates in females, which prevented wTei, wRi, and wMel from producing sufficient concentrations of resc factors. Excluding the study by Zabalou *et al.* [19] would not substantially alter the verdict concerning either of the two models.

### CI levels

We tested the following predictions: 1) more *Wolbachia* strains in females should decrease the CI level, 2) more *Wolbachia* strains in multiply infected males should increase the CI level, and 3) equivalent crosses should lead to similar CI levels. More details on the data set and the analytic method used for this section can be found in Text S1.

### Population genetics

We combined our findings on predicted CI patterns with population genetic simulations to test the models for plausibility and to make new predictions. Our first simulation aimed at analyzing the evolution of double-infections when the two strains involved are unidirectionally incompatible, that is, if one strain can rescue the other but not *vice versa*. We made the assumption that strains 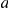 and 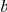 are transmitted with probability 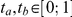, and that the double infection is transmitted with probability 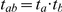. CI was simulated as causing embryonic mortality, allowing the percentage of unviable offspring in incompatible crosses to differ for mono or double infections. In addition, the host population was assumed to be panmictic, generations to be non-overlapping, and sex ratios to be equal. Differences in fecundity were excluded.

The second model was similar to the first except that double-infections were not allowed. For the goalkeeper-model, we assumed that the amount of mod and resc factors were within the boundary of 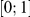 and that both factors triggered embryonic death independently from each other, with probability increasing linearly with the difference between mod and resc factor (if this difference was positive).

## Supporting Information

Text S1 Text S1 explains in more detail the formalism of the general framework used to study the goalkeeper-model, the lock-key-model, and the mistiming-model. Additional predictions derived from the models are presented, as well as proofs for all statements. Moreover, the tentative tests on CI levels are explained in more detail.
(PDF)
