# Supplementary material for: A New Model and Method for Understanding Wolbachia-Induced Cytoplasmic Incompatibility
Source: PLoS One. 2011 May 10;6(5):e19757. doi: 10.1371/journal.pone.0019757 (PMC3091874; doi:10.1371/journal.pone.0019757)
Supplement: Text S1 — explains in more detail the formalism of the general framework used to study the goalkeeper-model, the lock-key-model, and the mistiming-model. Additional predictions derived from the models are presented, as well as proofs for all statements. Moreover, the tentative tests on CI levels are explained in more detail. (PDF) [file pone.0019757.s001.pdf]

S1: Supporting Text: A New Model and Method for understanding *Wolbachia*-induced Cytoplasmic Incompatibility

Benjamin Bossan[1,*], Arnulf Koehncke[1], Peter Hammerstein[1]
1 Institute for Theoretical Biology, Humboldt University, Berlin, Germany
∗ E-mail: benjamin.bossan@hu-berlin.de

Introduction

In the first section, we describe in more detail the general framework from which the diverse models were derived. In the second section, we explain the goalkeeper-model more carefully; further statements are derived and all proofs are given. In the third section, we explain the lock-key-model in more detail; further statements are derived, all proofs are given, and we show a summary comparison of the two models. According to the available data, both models could be correct. In the third section, we present the mistiming-model using our framework; we argue that even with modifications, this model is problematic with regard to certain data. In the fifth section, we describe in more detail how the predictions with regard to CI levels were derived and the data used to test the predictions.

1 Formalism of the models of CI occurrence

We develop a formalism for models of CI occurrence and introduce a number of terms and definitions:

- $F_{CI}(i;ii;iii;iv)$ is a model of CI mechanism. It has four distinct attributes (see below).
- Lowercase letters $a, b, c, \ldots$ (except h, x, y, z) are *Wolbachia* strains with $a, b, c, \ldots \in \mathbb{W}$ representing a subset of *Wolbachia* strains of the pool of all possible *Wolbachia* strains $\mathbb{W}$.
- Lowercase h are specific host species with $h_1, h_2, \ldots \in \mathbb{H}$ representing a set of hosts from the pool of all possible hosts $\mathbb{H}$.
- $x, y, z \ldots$ are different mod and resc factors with $x_a, y_a, \ldots, \in \mathbb{R}^+$ representing quantities of possible factors of the pool of all possible factors. Subscripts indicate which *Wolbachia* strain or host produces this factor. Factors can be possessed by several strains and hosts, and any strain or host can possess several factors.
- $x_a^{mod}, y_a^{mod}, \ldots$ and $x_a^{resc}, y_a^{resc}, \ldots$ are the factors a *Wolbachia* strain a contributes to modification and rescue, respectively. All such contributions by *Wolbachia* are defined as non-negative.
- $x_h^{mod}, x_h^{resc}, y_h^{mod}, y_h^{resc}, \ldots$ are the factors that hosts contribute to their own modification and rescue.
- *Wolbachia* strains $a, b, \ldots$ and the host h rescue *Wolbachia* strains $a', b', \ldots$ if and only if (assuming additivity of factors):
  $\forall h \forall x, y, z, \ldots : (x_a^{resc} + x_b^{resc} + \ldots + x_h^{resc} \geq x_{a'}^{mod} + x_{b'}^{mod} + \ldots + x_h^{mod}) \wedge (y_a^{resc} + y_b^{resc} + \ldots + y_h^{resc} \geq y_{a'}^{mod} + y_{b'}^{mod} + \ldots y_h^{mod}) \wedge \ldots$
- $x_h, y_h, \ldots$ represents the "net host contribution" of factors $x, y, \ldots$ such that:
  $\forall h \forall x, y, z, \ldots : x_h = x_h^{resc} - x_h^{mod}; y_h = y_h^{resc} - y_h^{mod}, \ldots$.
  Net host contributions are only defined if mated male and female belong to the same host strain since host contributions then are not expected to differ significantly among individuals of the tested population. Given that every host rescues itself in the control cross, i.e. without *Wolbachia* infection,

$\forall h \forall x, y, z, \ldots : x_h \geq 0; y_h \geq 0; \ldots$ holds. Hence, if net host contributions are defined, *Wolbachia* strains $a, b, c, \ldots$ and host h rescue *Wolbachia* strains $a', b', c', \ldots$ if and only if: $\forall h \forall x, y, z, \ldots$: $(x_a^{resc} + x_b^{resc} + \ldots + x_h \geq x_{a'}^{mod} + x_{b'}^{mod} + \ldots) \wedge (y_a^{resc} + y_b^{resc} + \ldots + y_h \geq y_{a'}^{mod} + y_{b'}^{mod} + \ldots) \wedge \ldots$.

A model of CI mechanisms $F_{CI}(i; ii; iii; iv)$ can have the following attributes:

- i: Shows the number of mod/resc factors included in the model; $i \in \mathbb{N}$.
- ii: Shows whether mod and resc are quantitatively identical for their corresponding factor: $\forall a \forall x, y, z, \ldots$: $x_a^{mod} = x_a^{resc}, y_a^{mod} = y_a^{resc}, \ldots$, then $ii = 0$, or else $ii = 1$.
  If mod and resc are identical, then they may be expressed by the same genes or controlled by the same promoter. Otherwise, they probably are not.
- iii: Shows whether mod and resc factors are distributed on all $\mathbb{R}^+$, then $iii = 1$, or are quasi binary: $\forall a \forall x, y, z, \ldots : x_a^{mod} = \{0; 1\}, x_a^{resc} = \{0; \infty\}, y_a^{mod} = \{0; 1\}, y_a^{resc} = \{0; \infty\}, \ldots$, then $iii = 0$.
  This attribute examines the importance of quantitative differences. If factors are quasi binary, then presence or absence of the corresponding resc factor determines CI, irrespective of quantities. This may be the case when resc factor quantities are always much greater than mod factor quantities. Of course, factor quantities could also take neither of the proposed forms. If they were positive integers, for example, then iii would be between 0 and 1.
- iv: Shows whether net host contribution is present, then $iv = 1$, or absent (i.e. $\forall h \forall x, y, z, \ldots : x_h = 0; y_h = 0; \ldots$), then $iv = 0$.
  The latter case may be due to *Wolbachia* contributions exceeding those of the host, so that net host contributions, even if present, are negligible.

A model $F_{CI}^{(1)}(i; ii; iii; iv)$ is strictly more parsimonious than a model $F_{CI}^{(2)}(i'; ii'; iii'; iv')$ if and only if condition (A) is met: $i \leq i', ii \leq ii', iii \leq iii', iv \leq iv'$. If (A) is true, $F_{CI}^{(1)}$ has a higher falsifiability than $F_{CI}^{(2)}$; it is a special case of $F_{CI}^{(2)}$. Simply put, if a statement can be derived with $i = n$ factors, it can also be derived with $i = n + 1$ factors; identical mod and resc in females and males are a special case of them not necessarily being identical; quasi-binary factor quantities are a special case of factors being positive real numbers; and zero not host contribution is a special case of any non-negative net host contribution. Of two unfalsified models, the one with higher falsifiability should be preferred. However, no statement on falsifiability can be made if condition (A) is not met. For example, one cannot say whether the goalkeeper-model or the lock-key-model has a higher falsifiability than the other.

2 Goalkeeper-model

2.1 Presentation of the goalkeeper-model

The goalkeeper-model is a $F_{CI}(2; 0; 1; 1)$ model of CI: it takes into account two factors $x, y \in \mathbb{R}^+$; mod and resc function are quantitatively identical ($x^{mod} = x^{resc}, y^{mod} = y^{resc}$), and there is a net host contribution $x_h, y_h \geq 0$.

Here is how the model treats the four basic crosses that are possible:

- In the control cross (both parents uninfected), incompatibility does not occur if $x_h^{resc} - x_h^{mod} \geq 0 \Leftrightarrow x_h \geq 0$ and $y_h^{resc} - y_h^{mod} \geq 0 \Leftrightarrow y_h \geq 0$. Since these conditions are necessary for uninfected hosts to rescue themselves, we suppose them to always be true.
- In the reverse cross (only the female infected), CI does not occur if $x_h + x_a \geq 0$ and $y_h + y_a \geq 0$. Since the left hand quantities are always positive or zero, their sums are also positive or zero. Therefore, these conditions hold irrespective of the *Wolbachia* strain.

- In the compatible cross (both parents infected by the same strain), the female produces $x_h^{resc} + x_a$ and $y_h^{resc} + y_a$ and the male produces $x_h^{mod} + x_a$ and $y_h^{mod} + y_a$. Thus, CI does not occur if $x_h^{resc} + x_a \geq x_h^{mod} + x_a \Leftrightarrow x_h \geq 0$ and $y_h^{resc} + y_a \geq y_h^{mod} + y_a \Leftrightarrow y_h \geq 0$. This is the same condition as in the control cross and thus always true. Therefore, if both parents are infected by the same *Wolbachia* strain, CI never occurs.

- In the incompatible cross with only the male infected, the male produces $x_h^{mod} + x_a$ and $y_h^{mod} + y_a$ and the female produces x_h^{resc} and y_h^{resc}. CI does not occur if $x_h^{resc} \geq x_h^{mod} + x_a \Leftrightarrow x_h \geq x_a$ and $y_h^{resc} \geq y_h^{mod} + y_a \Leftrightarrow y_h \geq y_a$.

Since this analysis shows that x_h^{resc}, x_h^{mod}, y_h^{resc}, and y_h^{mod} are not explicitly required, we use the more convenient notion of net host contribution, x_h and y_h, instead.

The goalkeeper-model predicts CI never to occur in the control cross, compatible cross, and reverse cross. This complies with empirical observations. Conversely, while intuition suggests that incompatible crosses should always lead to CI, the model expects CI only to occur if the last condition is not met—i.e. if $x_a > x_h$, $y_a > y_h$, or both. However, what may be perceived as erroneous in the model can explain observations of "occasional" CI, i.e. when [mod-] *Wolbachia* strains that usually do not lead to CI unexpectedly induce CI in transfection experiments [1]. If we assume that host 1 produces x_{h1} and y_{h1}, and host 2 produces x_{h2} and y_{h2}, and further that quantities produced by host 1 are greater than those produced by host 2, then the last condition is met more easily in host 1 than in host 2. Therefore, the *Wolbachia* strain may induce CI in host 2 but not in host 1 (see also the proof in Table 16 on page 9).

The goalkeeper-model can be visualized by interpreting the two mod and resc factors as vectors in two-dimensional space (see figure 2 of the main text). CI does not occur if the sum of the mod vectors lies inside the area delimited by the resc vectors or if, vice versa, the sum of the resc vectors encloses the area delimited by the mod vectors. This graphic approach reaffirms that CI never occurs in the control, compatible, and reverse cross.

A goalkeeper-model without host contribution is possible (a $F_{CI}(2;0;1;0)$ model) and would be more parsimonious than the model presented so far. However, such a model would not allow for intransitivity (table 12) as well as for the additional statements M, N, and O (tables 16, 17, and 18). We therefore believe that host contribution as conceptualized here not only necessary but provides a useful framework to understand CI-related parasite-host interactions (as e.g. studied by [2]).

If a host is infected by several different *Wolbachia* strains simultaneously, we assume total factor quantity to be the sum of each individual strain's factors, i.e. without synergistic or antagonistic effects. This could be an oversimplification since theoretical considerations show that *Wolbachia* strains should reduce their replication rate when other strains are present [3]. However, empirical work showed that *Wolbachia* density is not strongly affected by the presence of additional *Wolbachia* strains [4,5], although a notable exception has been found [6]. Therefore, before relying too strictly on additivity, it should be tested whether *Wolbachia* density is affected in multiple infections and if it is, CI levels should be corrected for density effects. Still, as few exceptions to additivity have been observed, our model assumes for reasons of simplicity that factors act additively in multiple infections.

2.2 Formalism

We symbolize non-infection with the zero element, $\mathfrak{o}$. Further, we use the function R ("rescue") to write "*Wolbachia* strain a rescues *Wolbachia* strain b" as simply aRb. For two hosts h_i and h_j, a rescues b in host h_i is written as aR_ib, whereas a rescues b in host h_j is written as aR_jb.

2.3 Derivations: goalkeeper-model I

Table 1. Definitions

Reference	Definition
X	$\forall a, b, \ldots : a = (x_a, y_a), b = (x_b, y_b), \ldots x, y \in \mathbb{R}^+$, $\mathfrak{o} = (0, 0)$
Y	$\forall a, b \forall h : aRb \Leftrightarrow ((x_a + x_h \geq x_b) \wedge (y_a + y_h \geq y_b))$
Z	$\forall a, b : ab = (x_a + x_b, y_a + y_b)$

Table 2. Derivation of A

Statement: $\forall a \forall h : aR\mathfrak{o}$

	Statement	Reason
1	$\forall a \forall h : (x_h \geq 0) \wedge (y_h \geq 0)$	(X)
2	$(x_a \geq 0) \wedge (y_a \geq 0)$	(X)
3	$(x_a + x_h \geq 0) \wedge (y_a + y_h \geq 0)$	(1),(2), $\mathbb{R}$ is an ordered field
4	$aR\mathfrak{o}$	(3), (Y)

Table 3. Derivation of B

Statement: $\exists a, b \exists h : \neg (aRb) \wedge \neg (bRa)$

	Statement	Reason
1	$x_a = 2, y_a = 4, x_b = 4, y_b = 2, x_h = y_h = 1$	hypothesis
2	$\exists a, b \exists h : \neg((x_a + x_h \geq x_b) \wedge (y_a + y_h \geq y_b)) \wedge \neg((x_b + x_h \geq x_a) \wedge (y_b + y_h \geq y_a))$	(1)
3	$\neg (aRb) \wedge \neg (bRa)$	(2), (Y)

Table 4. Derivation of C

Statement: $\exists a, b \exists h : aRb \wedge \neg (bRa)$

	Statement	Reason
1	$x_a = y_a = 4, x_b = y_b = 2, x_h = y_h = 1$	hypothesis
2	$\exists a, b \exists h : ((x_a + x_h \geq x_b) \wedge (y_a + y_h \geq y_b)) \wedge \neg((x_b + x_h \geq x_a) \wedge (y_b + y_h \geq y_a))$	(1)
3	$aRb \wedge \neg (bRa)$	(2), (Y)

Table 5. Derivation of D

Statement: $\forall a, b, c \forall h : \neg(aRb) \Rightarrow \neg(aRbc)$

	Statement	Reason
1	$\forall a, b, c \forall h : aRbc$	hypothesis
2	$(x_a + x_h \geq x_b + x_c) \wedge (y_a + y_h \geq y_b + y_c)$	(1), (Y)
3	$\forall a, b, c : (x_b \geq 0) \wedge (y_b \geq 0) \wedge (x_c \geq 0) \wedge (y_c \geq 0)$	(X)
4	$(x_b + x_c \geq x_c) \wedge (y_b + y_c \geq y_c) \wedge (x_b + x_c \geq x_b) \wedge (y_b + y_c \geq y_b)$	(3), $\mathbb{R}$ is an ordered field
5	$(x_a + x_h \geq x_b) \wedge (y_a + y_h \geq y_b) \wedge (x_a + x_h \geq x_c) \wedge (y_a + y_h \geq y_c)$	(2), (4), transitivity of real numbers
6	$aRb \wedge aRc$	(5), (Y)
7	$\forall a, b, c \forall h : aRbc \Rightarrow (aRb \wedge aRc)$	(1), (6)
8	$\forall a, b, c \forall h : aRbc \Rightarrow aRb$	(7)
9	$\forall a, b, c \forall h : \neg(aRb) \Rightarrow \neg(aRbc)$	logical equivalence

Table 6. Derivation of D'

Statement: $\forall a, b \forall h : aRab \Rightarrow \mathfrak{o}Rb$

	Statement	Reason
1	$\forall a, b \forall h : aRab$	hypothesis
2	$\mathfrak{o}Rb$	(1), [K]
3	$aRab \Rightarrow \mathfrak{o}Rb$	(1), (2)

Table 7. D" is not true in this model

Statement: $\forall a, b \forall h : aRb \Rightarrow aRab$

	Statement	Reason
1	$\forall a, b \forall h : aRb \Rightarrow aRab$	indirect proof hypothesis
2	$x_a = y_a = 3, x_b = y_b = 2, x_h = y_h = 1$	hypothesis
3	$\exists a, b \exists h : (x_a + x_h \geq x_b) \wedge (y_a + y_h \geq y_b) \wedge \neg((x_a + x_h \geq x_a + x_b) \wedge (y_a + y_h \geq y_a + y_b))$	(2)
4	$aRb \wedge \neg aRab$	(3), (Y)
5	$\neg(\neg(aRb \wedge \neg(aRab)))$	logical equivalence
6	$\exists a, b \exists h : \neg(aRb \Rightarrow aRab)$	logical equivalence
7	contradiction (1) and (6)	$\forall X : \neg(X \wedge \neg X)$
8	$\exists a, b \exists h : \neg(aRb \Rightarrow aRab)$	(1) is false, its negation is true

Table 8. Derivation of E

Statement:$\forall a, b, c \forall h : aRb \Rightarrow acRb$

	Statement	Reason
1	$\exists a, b, c \exists h : \neg(aRb \Rightarrow acRb)$	indirect proof hypothesis
2	$\neg(\neg(aRb \wedge \neg acRb))$	logical equivalence
3	$aRb \wedge \neg acRb$	logical equivalence
4	$(x_a + x_h \geq x_b) \wedge (y_a + y_h \geq y_b) \wedge \neg((x_a + x_c + x_h \geq x_b) \wedge (y_a + y_c + y_h \geq y_b))$	(3), (Y)
5	$(x_a + x_h \geq x_b) \wedge (y_a + y_h \geq y_b) \wedge ((x_a + x_c + x_h < x_b) \vee (y_a + y_c + y_h < y_b))$	logical equivalence
6	$((x_a + x_h \geq x_b) \wedge (y_a + y_h \geq y_b) \wedge (x_a + x_c + x_h < x_b)) \vee ((x_a + x_h \geq x_b) \wedge (y_a + y_h \geq y_b) \wedge (y_a + y_c + y_h < y_b))$	logical equivalence
7	$(x_a + x_h \geq x_b > x_a + x_c + x_h) \vee (y_a + y_h \geq y_b > y_a + y_c + y_h)$	(6), $\mathbb{R}$ is an ordered field
8	$(0 > x_c) \vee (0 > y_c)$	(7), $\mathbb{R}$ is an ordered field
9	$(x_c \geq 0) \wedge (y_c \geq 0)$	(X)
10	8 is false	contradiction (8) and (9)
11	$\forall a, b, c \forall h : aRb \Rightarrow acRb$	(1) is false, its negation is true

Table 9. Derivation of E'

Statement: $\forall a, b \forall h : abRa$

	Statement	Reason
1	$\forall a, b \forall h : aRa$	[L]
2	$abRa$	[E]

Table 10. Derivation of F

Statement: $\exists a, b \exists h : \mathfrak{o}Ra \wedge \neg(\mathfrak{o}Rb) \wedge aRb$

	Statement	Reason
1	$x_a = y_a = 3, x_b = y_b = 6, x_h = y_h = 4$	hypothesis
2	$\exists a, b \exists h : ((x_h \geq x_a) \wedge (y_h \geq y_a)) \wedge \neg((x_h \geq x_b) \wedge (y_h \geq y_b)) \wedge ((x_a + x_h \geq x_b) \wedge (y_a + y_h \geq y_b))$	(1)
3	$\mathfrak{o}Ra \wedge \neg(\mathfrak{o}Rb) \wedge aRb$	(2), (Y)

2.4 Derivations: goalkeeper-model II

Table 11. Derivation of H

Statement: $\exists a, b, c \exists h : \neg(bRa) \wedge \neg(cRa) \wedge bcRa$

	Statement	Reason
1	$x_a = y_a = 5, x_b = y_b = x_c = y_c = x_h = y_h = 2$	hypothesis
2	$\exists a, b, c \exists h : \neg((x_b + x_h \geq x_a) \wedge (y_b + y_h \geq y_h)) \wedge\neg((x_c + x_h \geq x_a) \wedge (y_c + y_h \geq y_a))\wedge ((x_b+x_c+x_h \geq x_a)\wedge(y_b+y_c+y_h \geq y_a))$	(1)
3	$\neg(bRa) \wedge \neg(cRa) \wedge bcRa$	(2), (Y)

Table 12. Derivation of I

Statement: $\exists a, b, c \exists h : aRb \wedge bRc \wedge \neg(aRc)$

	Statement	Reason
1	$x_a = 1, y_a = 5, x_b = y_b = 3, x_c = 5, y_c = 1, x_h = 3, y_h = 1$	hypothesis
2	$\exists a, b, c \exists h : ((x_a + x_h \geq x_b) \wedge (y_a + y_h \geq y_c)) \wedge((x_b + x_h \geq x_c) \wedge (y_b + y_h \geq y_c))\wedge \neg((x_a + x_h \geq x_c) \wedge (y_a + y_h \geq y_c))$	(1)
3	$aRb \wedge bRc \wedge \neg(aRc)$	(2), (Y)

Table 13. J is not true in this model

Statement: $\forall a, b, c \forall h : aRbc \Rightarrow acRabc$

	Statement	Reason
1	$\forall a, b, c \forall h : aRbc \Rightarrow acRabc$	indirect proof hypothesis
2	$x_a = y_a = 3, x_b = 2, y_b = 1, x_c = 1, y_c = 2,\ x_h = y_h = 1$	hypothesis
3	$\exists a, b, c \exists h : ((x_a + x_h \geq x_b + x_c) \wedge (y_a + y_h \geq y_b + y_c)) \wedge \neg((x_h \geq x_b) \wedge (y_h \geq y_b))$	(2)
4	$aRbc \wedge \neg\, (\mathbf{o}Rb)$	(3), (Y)
5	$aRbc \wedge \neg\, (acRabc)$	(4), [K]
6	$\neg(\neg(aRbc \wedge \neg\, (acRabc)))$	logical equivalence
7	$\neg(aRbc \Rightarrow acRabc)$	logical equivalence
8	contradiction (1) and (7)	$\forall X : \neg(X \wedge \neg X)$
9	$\exists a, b, c \exists h : \neg(aRbc \Rightarrow acRabc)$	(1) is false, its negation is true

Table 14. Derivation of K

Statement:$\forall a, b, c \forall h : aRb \Leftrightarrow acRbc$

	Statement	Reason
1	$\forall a, b, c \forall h : aRb$	Hypothesis
2	$(x_a + x_h \geq x_b) \wedge (y_a + y_h \geq y_b)$	(1), (Y)
3	$(x_a + x_h + x_c \geq x_b + x_c) \wedge (y_a + y_h + y_c \geq y_b + y_c)$	$\mathbb{R}$ is an ordered field
4	$acRbc$	(3), (Y), (Z)
5	$aRb \Rightarrow acRbc$	(1), (4)
6	$\forall a, b, c \forall h : acRbc$	hypothesis
7	$(x_a + x_h + x_c \geq x_b + x_c) \wedge (y_a + y_h + y_c \geq y_b + y_c)$	(Y), (Z)
8	$(x_a + x_h \geq x_b) \wedge (y_a + y_h \geq y_b)$	$\mathbb{R}$ is an ordered field
9	aRb	(8), (Y)
10	$acRbc \Rightarrow aRb$	(6), (9)
11	$\forall a, b, c \forall h : (aRb \Rightarrow acRbc) \wedge (acRbc \Rightarrow aRb)$	(5), (10)
12	$aRb \Leftrightarrow acRbc$	logical equivalence

K' and K" are implied by K, and because K is true, K' and K" are also true.

Table 15. Derivation of L

Statement: $\forall a \forall h : aRa$

P	Statement	Reason
1	$\forall a \forall h : (x_h \geq 0) \wedge (y_h \geq 0)$	(X)
2	$(x_h + x_a \geq x_a) \wedge (y_h + y_a \geq y_a)$	$\mathbb{R}$ is an ordered field
3	aRa	(Y)

Table 16. Derivation of M

Statement: $\exists a \exists h_1, h_2 : \mathfrak{o}R_1 a \wedge \neg(\mathfrak{o}R_2 a)$

	Statement	Reason
1	$x_a = y_a = 2, x_{h_1} = y_{h_1} = 3,$<br>$x_{h_2} = y_{h_2} = 1$	hypothesis
2	$\exists a \exists h_1, h_2 : (x_{h_1} \geq x_a) \wedge (y_{h_1} \geq y_a) \wedge$<br>$\neg((x_{h_2} \geq x_a) \wedge (y_{h_2} \geq y_a))$	(1)
3	$\mathfrak{o}R_1 a \wedge \neg(\mathfrak{o}R_2 a)$	(2), (Y)

Table 17. Derivation of N

$\exists a, b \exists h_1, h_2 : \mathfrak{o}R_1 a \wedge \neg(\mathfrak{o}R_1 b) \wedge \neg(\mathfrak{o}R_2 a) \wedge \mathfrak{o}R_2 b$

	Statement	Reason
1	$x_a = 3, y_a = 1, x_b = 1, y_b = 3,$<br>$x_{h_1} = 4, y_{h_1} = 2, x_{h_2} = 2, y_{h_2} = 4$	hypothesis
2	$\exists a, b \exists h_1, h_2 : (x_{h_1} \geq x_a) \wedge (y_{h_1} \geq y_a) \wedge$<br>$\neg((x_{h_1} \geq x_b) \wedge (y_{h_1} \geq y_b)) \wedge$<br>$\neg((x_{h_2} \geq x_a) \wedge (y_{h_2} \geq y_a)) \wedge$<br>$(x_{h_2} \geq x_b) \wedge (y_{h_2} \geq y_b)$	(1)
3	$\mathfrak{o}R_1 a \wedge \neg(\mathfrak{o}R_1 b) \wedge \neg(\mathfrak{o}R_2 a) \wedge \mathfrak{o}R_2 b$	(2), (Y)

Table 18. Derivation of O

Statement: $\exists a, b \exists h_1, h_2 : aR_1 b \wedge \neg(aR_2 b)$

	Statement	Reason
1	$x_a = y_a = 2,\ x_b = y_b = 4,$<br>$x_{h_1} = y_{h_1} = 3,\ x_{h_2} = y_{h_2} = 1$	hypothesis
2	$\exists a \exists h_1, h_2 : (x_a + x_{h_1} \geq$<br>$x_b) \wedge (y_a + y_{h_2} \geq y_b)$<br>$\wedge \neg((x_a + x_{h_2} \geq x_b) \wedge (y_a + y_{h_2} \geq y_b))$	(1)
3	$aR_1 b \wedge \neg(aR_2 b)$	(2), (Y)

Table 19. P is not true in this model

Statement: $\forall a, b, c \forall h : ((aRb) \wedge (aRc)) \Rightarrow aRbc$

	Statement	Reason
1	$\forall a, b, c : ((aRb) \wedge (aRc)) \Rightarrow aRbc$	indirect proof hypothesis
2	$x_a = y_a = 4$, $x_b = y_b = 3$, $x_c = y_c = 3, x_h = y_h = 1$	hypothesis
3	$\exists a, b, c \exists h : (x_a + x_h \geq x_b) \wedge (y_a + y_h \geq y_b) \wedge (x_a + x_h \geq x_c) \wedge (y_a + y_h \geq y_c) \wedge (x_a + x_h < x_b + x_c) \wedge (y_a + y_h < y_b + y_c)$	(1)
4	$aRb \wedge aRc \wedge \neg (aRbc)$	(2), (Y)
5	contradiction (1) and (4)	$\forall X : \neg(X \wedge \neg X)$
6	$\exists a, b, c \exists h : \neg (((aRb) \wedge (aRc)) \Rightarrow aRbc)$	(1) is false, its negation is true

3 Lock-key-model

3.1 Presentation of the lock-key-model

The lock-key-model is a $F_{CI}(\infty; 1; 0; 0)$ model: It allows for an infinite number of mod and resc factors; mod and resc factors need not be identical and are called "locks" and "keys", respectively; quantitative differences *within* factors are ignored and only presence or absence is considered; host contributions are not assumed.

The lock-key-model explains bidirectional incompatibility by assuming every *Wolbachia* strain to have its own locks and keys. Explaining asymmetrical incompatibility is harder—either master keys exist that open several locks, or some *Wolbachia* strains have more keys than needed to open their own locks. Both assumptions produce similar results, but the latter is easier to handle and thus chosen here.

3.2 Formalism

Since CI is determined by whether the set of all locks can be matched by the set of all keys and not by quantitative questions, we use a different language than in the goalkeeper-model. We define the set of all locks of a *Wolbachia* strain a as L_a and the set of all keys as K_a. The locks and keys are called $x_1, x_2, \ldots$. Then, "a rescues b" (aRb) if and only if the set of all locks is a subset of the set of all keys. In multiple infections, the total factors provided are the union of the factors provided by each strain. All definitions and axioms necessary for deriving statements in the lock-key-model are given in Table 20 on page 11.

3.3 Derivations: lock-key-model I

Table 20. Definitions

Reference	Definition
T	$\forall a \neq \mathfrak{o} : L_a = \{x_i, x_j, \ldots\}, K_a = \{x_k, x_l, \ldots\}$
Reference	Axioms
U	$\forall a, b, i : (S_a \supseteq S_b) \Leftrightarrow ((x_i \in S_b) \Rightarrow (x_i \in S_a))$,$S_a = K_a \vee L_a, S_b = K_b \vee L_b$
V	$\exists \mathfrak{o} : \forall i, j : (\neg(x_i \in L_{\mathfrak{o}})) \wedge (\neg(x_j \in K_{\mathfrak{o}}))$, or $\exists \mathfrak{o} : (L_{\mathfrak{o}} = \varnothing) \wedge (K_{\mathfrak{o}} = \varnothing)$
W	$\forall a, b : (L_a \subseteq (L_a \cup S_b)) \wedge (K_a \subseteq (K_a \cup S_b))$
X	$\forall a, b : aRb \Leftrightarrow (K_a \supseteq L_b)$
Y	$\forall a : aRa$
Z	$\forall a, b : (L_{ab} = L_a \cup L_b), (K_{ab} = K_a \cup K_b)$

Table 21. Derivation of A

Statement: $\forall a : aR\mathfrak{o}$

	Statement	Reason
1	$\forall a : L_{\mathfrak{o}} = \varnothing$	(V)
2	$K_a \supseteq L_{\mathfrak{o}}$	(1), (T)
3	$aR\mathfrak{o}$	(X)

Table 22. Derivation of B

Statement: $\exists a, b : \neg(aRb) \wedge \neg(bRa)$

	Statement	Reason
1	$L_a = K_a = \{x_1\}, L_b = K_b = \{x_2\}$	hypothesis
2	$\exists a, b : \neg(K_a \supseteq L_b) \wedge \neg(K_b \supseteq L_a)$	(1), (U)
3	$\neg\,(aRb) \wedge \neg\,(bRa)$	(X)

Table 23. Derivation of C

Statement: $\exists a, b : aRb \wedge \neg\,(bRa)$

	Statement	Reason
1	$L_a = K_a = \{x_1, x_2\}, L_b = K_b = \{x_2\}$	hypothesis
2	$\exists a, b : (K_a \supseteq L_b) \wedge \neg(K_b \supseteq L_a)$	(1), (U)
3	$aRb \wedge \neg\,(bRa)$	(X)

Table 24. Derivation of D

Statement: $\forall a, b, c : \neg(aRb) \Rightarrow \neg(aRbc)$

	Statement	Reason
1	$\forall a, b, c : aRbc$	hypothesis
2	$K_a \supseteq L_{bc}$	(1), (X)
3	$K_a \supseteq (L_b \cup L_c)$	(2), (Z)
4	$(K_a \supseteq L_b) \wedge (K_a \supseteq L_c)$	logical equivalence
5	$aRb \wedge aRc$	(4), (X)
6	$aRbc \Rightarrow (aRb \wedge aRc)$	(1), (5)
7	$\forall a, b, c : aRbc \Rightarrow aRb$	(6)
8	$\forall a, b, c : \neg(aRb) \Rightarrow \neg(aRbc)$	logical equivalence

Table 25. D' is not true in this model

Statement: $\forall a, b : aRab \Rightarrow \mathfrak{o}Rb$

	Statement	Reason
1	$\forall a, b : aRab \Rightarrow \mathfrak{o}Rb$	indirect proof hypothesis
2	$L_a = K_a = \{x_1, x_2\}, L_b = K_b = \{x_1\}$	hypothesis
3	$\exists a, b : (K_a \supseteq L_b) \wedge (K_a \supseteq L_a) \wedge \neg(\varnothing \supseteq L_b)$	(3), (U)
4	$(K_a \supseteq (L_a \cup L_b)) \wedge \neg(\varnothing \supseteq L_b)$	(U), (Z)
5	$(K_a \supseteq L_{ab}) \wedge \neg(\varnothing \supseteq L_b)$	(4), (Z)
6	$aRab \wedge \neg(\mathfrak{o}Rb)$	(V), (X)
7	$\neg(\neg(aRab \wedge \neg(\mathfrak{o}Rb)))$	logical equivalence
8	$\neg(aRab \Rightarrow \mathfrak{o}Rb)$	logical equivalence
9	contradiction (1) and (8)	$\forall X : \neg(X \wedge \neg X)$
10	$\exists a, b, h : \neg(aRab \Rightarrow \mathfrak{o}Rb)$	(1) is false, its negation is true

Table 26. Derivation of D”

Statement: $\forall a, b : aRb \Rightarrow aRab$

	Statement	Reason
1	$\forall a, b : aRb$	hypothesis
2	$K_a \supseteq L_b$	(1), (X)
3	aRa	(Y)
4	$K_a \supseteq L_a$	(3), (X)
5	$(K_a \supseteq L_a) \wedge (K_a \supseteq L_b)$	(4), (2)
6	$K_a \supseteq (L_a \cup L_b)$	logical equivalence
7	$K_a \supseteq L_{ab}$	(6), (Z)
8	$aRab$	(7), (X)
9	$\forall a, b : aRb \Rightarrow aRab$	(1), (8)

Table 27. Derivation of E

Statement: $\forall a, b, c : aRb \Rightarrow acRb$

	Statement	Reason
1	$\exists a, b, c : \neg(aRb \Rightarrow acRb)$	indirect proof hypothesis
2	$\neg((K_a \supseteq L_b) \Rightarrow (K_{ac} \supseteq L_b))$	(1), (X)
3	$\neg(\neg((K_a \supseteq L_b) \wedge \neg(K_{ac} \supseteq L_b)))$	logical equivalence
4	$(K_a \supseteq L_b) \wedge \neg(K_{ac} \supseteq L_b)$	logical equivalence
5	$(K_a \cup K_c) \supseteq K_a$	(W)
6	$K_{ac} \supseteq K_a$	(5), (Z)
7	$K_{ac} \supseteq L_b$	(4), (6), (U)
8	$(K_a \supseteq L_b) \wedge \neg(K_a \supseteq L_b)$	(4), (7)
9	contradiction (8)	$\forall X : \neg(X \wedge \neg X)$
10	$\forall a, b : aRb \Rightarrow acRb$	(1) is false, its negation is true

Table 28. Derivation of E'

Statement: $\forall a, b : abRa$

	Statement	Reason
1	$\forall a, b : aRa$	(Y)
2	$abRa$	(1), [E]

Table 29. Derivation of F

Statement: $\exists a, b : \mathfrak{o}Ra \wedge \neg(\mathfrak{o}Rb) \wedge aRb$

	Statement	Reason
1	$L_a = \varnothing, K_a = \{x_1\}, L_b = K_b = \{x_1\}$	hypothesis
2	$(K_{\mathfrak{o}} \supseteq L_a) \wedge \neg(K_{\mathfrak{o}} \supseteq L_b) \wedge (K_a \supseteq L_b)$	(U), (V), (W)
3	$\mathfrak{o}Ra \wedge \neg(\mathfrak{o}Rb) \wedge aRb$	(2), (X)

3.4 Derivations: lock-key-model II

Table 30. Derivation of H

Statement: $\exists a, b, c : \neg(bRa) \wedge \neg(cRa) \wedge bcRa$

	Statement	Reason
1	$L_a = K_a = \{x_1, x_2\}, L_b = K_b = \{x_1\}, L_c = K_c = \{x_2\}$	hypothesis
2	$\exists a, b, c : \neg(K_b \supseteq L_a) \wedge \neg(K_c \supseteq L_a) \wedge (L_a \supseteq (K_b \cup K_c))$	(1), (U)
3	$\neg(L_a \supseteq K_b) \wedge \neg(L_a \supseteq K_c) \wedge (L_a \supseteq K_{bc})$	(2), (Z)
4	$\neg(bRa) \wedge \neg(cRa) \wedge bcRa$	(X)

Table 31. Derivation of I

Statement: $\exists a, b, c : aRb \wedge bRc \wedge \neg(aRc)$

	Statement	Reason
1	$L_a = \{x_1\}, K_a = \{x_1, x_2\}, L_b = \{x_2\}, K_b = \{x_1, x_2, x_3\}, K_c = L_c = \{x_3\}$	hypothesis
2	$\exists a, b, c : (K_a \supseteq L_b) \wedge (K_b \supseteq L_c) \wedge \neg(K_a \supseteq L_c)$	(2), (U)
3	$aRb \wedge bRc \wedge \neg(aRc)$	(X)

Table 32. Derivation of J

Statement: $\forall a, b, c : aRbc \Rightarrow acRabc$

	Statement	Reason
1	$\forall a, b, c : aRbc$	hypothesis
2	$acRbcc$	(1), [K]
3	$acRbc$	(2), (U)
4	$aacRabc$	(3), [K]
5	$acRabc$	(4), (U)
6	$\forall a, b, c : aRbc \Rightarrow acRabc$	(1), (5)

Table 33. Derivation of K'

Statement: $\forall a, b, c : aRb \Rightarrow acRbc$

	Statement	Reason
1	$\forall a, b, c : cRc$	(Y)
2	$K_c \supseteq L_c$	(2), (X)
3	$(K_a \cup K_c) \supseteq L_c$	(3), (W)
4	$K_{ac} \supseteq L_c$	(4), (Z)
5	$\forall a, b, c : aRb$	hypothesis
6	$K_a \supseteq L_b$	(X)
7	$(K_a \cup K_c) \supseteq L_b$	(6), (W)
8	$K_{ac} \supseteq L_b$	(7), (Z)
9	$\forall a, b, c : aRb \Rightarrow ((K_{ac} \supseteq L_b) \wedge (K_{ac} \supseteq L_c))$	(8), (4)
10	$aRb \Rightarrow (K_{ac} \supseteq (L_b \cup L_c))$	(9), (U)
11	$aRb \Rightarrow (K_{ac} \supseteq L_{bc})$	logical equivalence
12	$aRb \Rightarrow acRbc$	(11), (X)

Table 34. K", the converse of K', is not true in this model

Statement: $\forall a, b, c : acRbc \Rightarrow aRb$

	Statement	Reason
1	$acRbc \Rightarrow aRb$	indirect proof hypothesis
2	$L_a = K_a = \{x_1\}, L_b = K_b = \{x_1, x_2\}, L_c = K_c = \{x_2\}$	hypothesis
3	$\exists a, b, c : (((K_a \cup K_c) \supseteq L_b) \wedge ((K_a \cup K_c) \supseteq L_c) \wedge \neg(K_a \supseteq L_b)$	(2), (U)
4	$((K_a \cup K_c) \supseteq (L_b \cup L_c)) \wedge \neg(K_a \supseteq L_b)$	logical equivalence
5	$(K_{ac} \supseteq L_{bc}) \wedge \neg(K_a \supseteq L_b)$	(4), (Z)
6	$acRbc \wedge \neg\,(aRb)$	(5), (X)
7	$\neg(acRbc \Rightarrow aRb)$	logical equivalence
8	contradiction (1) and (7)	$\forall X : \neg(X \wedge \neg X)$
9	$\exists a, b, c : \neg(acRbc \Rightarrow aRb)$	(1) is false, its negation is true

Table 35. K is not true in this model

Statement: $\forall a, b, c : aRb \Leftrightarrow acRbc$

	Statement	Reason
1	$\forall a, b, c : aRb \Leftrightarrow acRbc$	indirect proof hypothesis
2	$(aRb \Rightarrow acRbc) \wedge (acRbc \Rightarrow aRb)$	logical equivalence
3	$\exists a, b, c : \neg(acRbc \Rightarrow aRb)$	[K"]
4	contradiction (2) and (3)	$\forall X : \neg(X \wedge \neg X)$
5	$\exists a, b, c : \neg(aRb \Leftrightarrow acRbc)$	(1) is false, its negation is true

Table 36. Derivation of P

Statement: $\forall a, b, c : ((aRb) \wedge (aRc)) \Rightarrow aRbc$

	Statement	Reason
1	$\forall a, b, c : (aRb) \wedge (aRc)$	hypothesis
2	$(K_a \supseteq L_b) \wedge (K_a \supseteq L_c)$	(1), (X)
3	$K_a \supseteq (L_b \cup L_C)$	logical equivalence
4	$K_a \supseteq L_{bc}$	(3), (Z)
5	$aRbc$	(4), (X)
6	$\forall a, b, c : ((aRb) \wedge (aRc)) \Rightarrow aRbc$	(1), (5)

3.5 Synopsis

Table 37. Summary of derived statements

	Table	goalkeeper	lock-key	test
A: $\forall a(\forall h) : aR\mathsf{o}$	2,21	true	true	evidence
B: $\exists a, b(\exists h) : \neg(aRb) \wedge \neg(bRa)$	3,22	true	true	proved
C: $\exists a, b(\exists h) : aRb \wedge \neg(bRa)$	4,23	true	true	proved
D: $\forall a, b, c(\forall h) : \neg(aRb) \Rightarrow \neg(aRbc)$	5,24	true	true	evidence
D': $\forall a, b(\forall h) : aRab \Rightarrow \mathsf{o}Rb$	6,25	true	false	not tested
D": $\forall a, b(\forall h) : aRb \Rightarrow aRab$	7,26	false	true	not tested
E: $\forall a, b, c(\forall h) : aRb \Rightarrow acRb$	8,27	true	true	evidence
E': $\forall a, b(\forall h) : abRa$	9,28	true	true	evidence
F: $\exists a, b(\exists h) : \mathsf{o}Ra \wedge \neg(\mathsf{o}Rb) \wedge aRb$	10,29	true	true	proved
H: $\exists a, b, c(\exists h) : \neg(bRa) \wedge \neg(cRa) \wedge bcRa$	11,30	true	true	not tested
I: $\exists a, b, c(\exists h) : aRb \wedge bRc \wedge \neg(aRc)$	12,31	true	true	proved
J: $\forall a, b, c(\forall h) : aRbc \Rightarrow acRabc$	13,32	false	true	not tested
K: $\forall a, b, c(\forall h) : aRb \Leftrightarrow acRbc$	14,34	true	false	not tested
K': $\forall a, b, c(\forall h) : aRb \Rightarrow acRbc$	14,33	true	true	not tested
K": $\forall a, b, c(\forall h) : acRbc \Rightarrow aRb$	14,34	true	false	not tested
L: $\forall a \forall h : aRa$	15,20	true	axiom	evidence
M: $\exists a \exists h_1, h_2 : \mathsf{o}R_1a \wedge \neg(\mathsf{o}R_2a)$	16	true	-	proved
N:				
$\exists a, b \exists h_1, h_2 : \mathsf{o}R_1a \wedge \neg(\mathsf{o}R_1b) \wedge \neg(\mathsf{o}R_2a) \wedge \mathsf{o}R_2b$	17	true	-	not found
O: $\exists a, b \exists h_1, h_2 : aR_1b \wedge \neg(aR_2b)$	18	true	-	not found
P: $\forall a, b, c\,(\forall h) : ((aRb) \wedge (aRc)) \Rightarrow aRbc$	19,36	false	true	not tested

4 Why the mistiming-model is most likely invalid

The mistiming-model assumes *Wolbachia* to manipulate sperm content to delay the male pronucleus during the first cell cycle after fertilization. Rescue restores synchrony by applying the same manipulation to the rest of the ovum, thus delaying it by the same degree [7,8]. The mistiming-model is a $F_{CI}(1;0;1;1)$ model, similar to the goalkeeper-model but with only one factor (we assume a net host contribution to make the model harder to falsify). Definitions are given in table 39.

The mistiming-model is a special case of the goalkeeper-model and has thus more predictive power. However, the mistiming-model cannot account for bidirectional incompatibility (proof see Table 40 on page 21).

Modifying the mistiming-model by assuming different resc factors in the ovum to bind to paternal chromosomes and further slow down their progression allows bidirectional incompatibility to occur [9]. However, this enlarged model can no longer explain unidirectional incompatibility between different *Wolbachia* strains because sperm would always be further delayed upon entering an ovum containing another strain. Assuming different binding sites for different factors can solve this new problem ([9]; see figure 1) but the model loses predictive power as a consequence.

The modified mistiming-model relies on the assumption that the sperm DNA can be further modified after fertilization. This implies that it is also modified by the factors contributed naturally by the mother, the host contribution to mod. As a consequence, we will momentarily drop the assumption that there is a net host contribution. The mistiming-model is thus altered to be an $F_{CI}(\infty;0;1;0)$ model of CI. The new definitions of the model can be found in Table 41 on page 20. In this model, a *Wolbachia* strain a can only rescue strain b if b does not produce factors binding to a site that the factors produced by a

Table 38. Verbal interpretation of all statements

Statement	Interpretation
A	If only in the ovum but not in the sperm, *Wolbachia* does not cause CI
B	Bidirectional incompatibility is possible
C	Unidirectional incompatibility is possible
D	Additional strains in males cannot decrease mod strength
D'	Even if a rescues b, it cannot rescue the double-infection ab (except if b is [mod-])
D"	If a rescues b, it also rescues the double-infection ab
E	Additional strains in females cannot decrease resc strength
E'	The double-infection ab rescues the mono-infection a
F	The existence of [mod- resc+] strains is possible
H	There are strains b and c that cannot rescue a by themselves but can do so together
I	Intransitivity – there is a strain a that rescues a strain b which can rescue c, but a cannot rescue c
J	If a rescues the double-infection bc, then the double infection ac rescues the triple-infection abc
K	Only if a rescues b does the double-infection ac rescue the double-infection bc
K'	If a rescues b, the double-infection ac rescues the double-infection bc
K"	If the double-infection ac rescues the double-infection bc, a rescues b
L	Each strain possesses the factors required to rescue itself
M	It is possible that a strain is [mod-] in one host and [mod+] in another
N	It is possible that in one host, a is [mod-] and b is [mod+], while the reverse is true in another host
O	It is possible that a strain a can rescue b in one host but not in another
P	If a rescues b and if a rescues c, it also rescues the double-infection bc

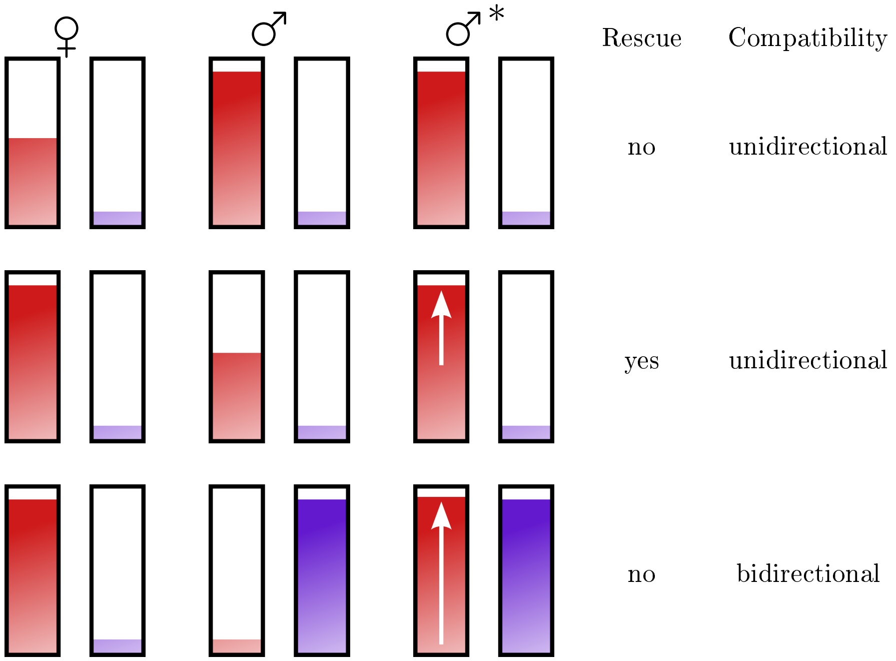

Figure 1. Different crosses of two Wolbachia strains in the modified mistiming-model. Red and blue bars represent quantities of two factors binding to two different sites. The first row depicts factor quantities in the the ovum, the second in the sperm. Upon fertilization, factors deposited in the ovum bind to the sperm DNA if the corresponding site is not already fully occupied (third row, white arrow). Rescue occurs if, after fertilization, none of the binding sites of the paternal DNA binds more factors than the binding sites of the maternal DNA.

do not bind to. In addition, b must not produce a higher quantity than a of any factor at a common binding site, leading to definition Y'. This modified mistiming-model can account for both bidirectional incompatibility and unidirectional incompatibility (figure 1, formal proof not shown).

Table 41. Definitions modified mistiming-model

Reference	Definition
X'	$\forall a, b, \ldots : a = (x_a, y_a, ...)\,, b = (x_b, y_b, ...)\,, \ldots x, y, \ldots \in \mathbb{R}^+$, $\mathfrak{o} = (0, 0, \ldots)$
Y'	$\forall a, b \forall h : aRb \Leftrightarrow ((x_a \geq x_b) \wedge (y_a \geq y_b) \wedge ...)$
Z'	$\forall a, b : ab = (\max\{x_a; x_b\}\,; \max\{y_a; y_b\}\,; ...)$

However, in contrast to both goalkeeper-model and lock-key-model, the modified mistiming-model predicts transitivity (i.e. when strain a rescues strain b, and strain b rescues strain c, then strain a must also rescue strain c; statement I in table 42). However, such transitivity is contradicted by empirical findings [1]. Again, we could add further ad hoc hypotheses, for example by making the mistiming-model an $F_{CI}(\infty, 0, 1, 1)$ model (the same as the previous but with net host contributions). However, this model would be strictly less parsimonious than the goalkeeper-model. We thus prefer to use the goalkeeper-model or the lock-key-model instead to explain CI.

5 Predictions from the norm approach to CI levels in the goalkeeper-model

5.1 Introduction

The goalkeeper-model does not contain a specific mechanism to explain CI levels. Still, some simple predictions can be derived from the intuitive reasoning that CI levels are proportional to the norm of the differences between mod and resc vector:

1. More *Wolbachia* strains in females should decrease CI levels due to the increased amount of resc factors.
2. More *Wolbachia* strains in males should increase CI levels due to the increased amount of mod factors.
3. Equivalent crosses, i.e. crosses in which the number of excess *Wolbachia* strains in the female or male are equal, should lead to similar CI levels because the difference in mod and resc factors should be equal.

Table 39. Definitions mistiming-model

Reference	Definition
X	$\forall a, b, \ldots \neq \mathfrak{o} : a = x_a, b = x_b, \ldots x \in \mathbb{R}^+$, $\mathfrak{o} = 0$
Y	$\forall a, b \forall h : aRb \Leftrightarrow (x_a + x_h \geq x_b)$
Z	$\forall a, b : ab = (x_a + x_b)$

Table 40. B is not true in the mistiming-model

Statement: $\exists a, b \exists h : \neg(aRb) \wedge \neg(bRa)$

	Statement	Reason
1	$\exists a, b \exists h : \neg(aRb) \wedge \neg(bRa)$	indirect proof hypothesis
2	$\neg(x_a + x_h \geq x_b) \wedge \neg(x_b + x_h \geq x_a)$	(1), (Y)
3	$(x_a + x_h < x_b) \wedge (x_b + x_h < x_a)$	logical equivalence
4	$(x_h < x_b - x_a) \wedge (x_h < x_a - x_b)$	(3), $\mathbb{R}$ is an ordered field
5	$(x_a - x_b < x_b - x_a) \wedge (x_b - x_a < x_a - x_b)$	Transitivity of real numbers
6	$(2 \cdot x_a < 2 \cdot x_b) \wedge (2 \cdot x_b < 2 \cdot x_a)$	(5), $\mathbb{R}$ is an ordered field
7	$\neg \exists a, b \neg \exists h : \neg(aRb) \wedge \neg(bRa)$	(6) is a contradiction, thus (1) is false

(To form an intuition of the reason for the three predictions, have a look at figure 2 of the main text and think of the CI level as proportional to the norm of the differences in the mod and the resc vector.)

To study these predictions, we only compared crosses of equal type, that is, compatible with compatible crosses, incompatible with incompatible crosses, etc. We excluded comparisons of different crossing types, like compatible vs incompatible cross or compatible vs control cross. For example, we tested whether females infected by wRi are better at rescuing crosses with males infected with wNo than uninfected females are (prediction 1). However, we do not consider that females infected with wNo are better at rescuing crosses with males infected by wNo than uninfected females are, because in this case, the additional *Wolbachia* strain in the female renders the cross compatible, whereas in the former case, both crosses are incompatible. Similarly, we tested for example whether males double-infected with wHa and wNo cause higher levels of CI when mated with uninfected females than males mono-infected with wHa or wNo do (prediction 2). Our predictions were agnostic as to whether, for example, the double-infection of wHa and wNo or whether the mono-infection of wRi should lead to higher CI levels when females are uninfected.

We define equivalent crosses as those crosses in which the number of excess *Wolbachia* strains in the female or male are equal. For example in the cross of uninfected females with wHa infected males, the wHa strain is in excess in males (it is not present in the females). Likewise, in the cross of wNo infected females with wHa and wNo double-infected males, the wHa strain is in excess in males (the wNo strain is present in both sexes, but only the wHa strain is present in males). That these crosses should be equivalent can be derived from statement K (table 14). As more precise statistical analyses were not possible from the given data, we treated CI levels as similar if the difference between the CI levels of all equivalent crosses was less than 10%.

Table 42. The negation of I is true in this model

Statement: $\forall a, b, c : (aRb \wedge bRc) \Rightarrow aRc$

	Statement	Reason
1	$\forall a, b, c : (aRb \wedge bRc)$	hypothesis
2	$(x_a > x_b) \wedge (y_a > y_b) \wedge ...$	(1), (Y')
3	$(x_b > x_c) \wedge (y_b > y_c) \wedge ...$	(1), (Y')
4	$(x_a > x_c) \wedge (y_a > y_c) \wedge ...$	(2), (3), transitivity of real numbers
5	$\forall a, b, c : aRc$	(4), (Y')

Table 43. CI levels (uncorrected) from crossing experiments from [10]. Rows: females, columns: males.

		♂				
		uninf.	wRi	wHa	wNo	wHa+wNo
	uninf.	0.270	0.990	0.973	0.812	0.992
	wRi	0.040	0.157	0.892	0.785	0.973
♀	wHa	0.037	0.983	0.107	0.786	0.651
	wNo	0.264	0.924	0.940	0.333	0.938
	wHa+wNo	0.058	0.992	0.130	0.093	0.194

Table 44. CI levels (uncorrected) from crossing experiments from [11]. Rows: females, columns: males.

		♂		
		wRi	wHa	wHa+wRi
	wRi	0.216	1.000	0.975
♀	wHa	0.992	0.190	0.991
	wHa+wRi	0.088	0.130	0.208

5.2 Method

Data were taken from [10] (their table 4), [11] (only controlled data, top of their table 1), and [12] their tables 2 and 3). If more than one data set for a cross was available within an article, we took the arithmetic mean of the data sets and weighted with number of observations, if indicated. Our accumulated data can be found in table 43, 44, and 45.

For example, prediction 1 could be tested as follows: Given males triple-infected with wHa, wRi, wNo, compare the CI levels of uninfected females (should be highest) with the CI level of females mono-infected by only one of those three strains (should be high but lower), and with the CI level of females double-infected by two of the three strains (should still be high but even lower). Thus, given the data of [12] (1999; table 45), we can test eight predictions of type 1 by analyzing the last column. Each possible comparison is counted as an individual test, draws were excluded. Because the data presented in the articles do not allow to perform statistical tests of significance, such tests were omitted. We merely tested whether the predictions we made were qualitatively correct.

5.3 Results

Of the predictions of type 1, 21 were correct and 4 were false (highly significant; $p<0.001$, one-tailed binomial test). Of the predictions of type 2, 14 were correct and 8 were false (1 draw excluded, difference

Table 45. CI levels (uncorrected) from crossing experiments from [12]. Rows: females, columns: males.

		♂						
		uninf.	wRi	wHa	wNo	wHa+wRi	wHa+wNo	wHa+wRi+wNo
	uninf.	0.089	0.852	0.766	0.996	-	0.852	0.996
	wRi	-	-	-	-	-	-	-
	wHa	-	-	-	-	-	-	0.991
♀	wNo	-	-	-	-	-	-	0.994
	wHa+wRi	-	-	-	-	0.317	0.604	0.800
	wHa+wNo	-	-	-	-	0.989	0.361	0.981
	wHa+wRi+wNo	0.070	-	-	0.185	0.275	0.230	0.393

insignificant). Of the predictions of type 3, 10 were correct and 3 were false (difference insignificant). Data can also be pooled by publication — [10]: 18 predictions were correct and 11 predictions were false (not significant); [11]: 5 predictions were correct and 2 predictions were false (not significant); [12]: 22 predictions were correct and 2 predictions were false ($p<0.001$, one-tailed binomial test, 1 draw excluded). Overall, 45 predictions were correct and 15 were false (1 draw excluded; highly significant; $p<0.001$, one-tailed binomial test).

5.4 Discussion

We analyzed three predictions made by the norm approach to CI levels within the framework of the goalkeeper-model: (1) more *Wolbachia* strains in females should decrease CI levels, (2) more *Wolbachia* strains in males should increase CI levels, and (3) equivalent crosses should yield similar CI levels. Data from three publications generated 60 predictions of which 45 were correct and 14 false (1 draw). Predictions of type 1 were correct significantly more often than expected by chance; predictions of types 2 and 3 were correct more often than not, but the differences were not significant, maybe owing to small sample sizes.

The norm approach can further be corroborated by looking at the results from [13]. By using predictions of type 3, one would not expect significant differences in CI levels *within* the incompatible crosses of

- type [a]: (♀+ wLhet1)×(♂+ wLhet1 + <u>wLhet3</u>) and (♀+ wLhet1 + wLhet2)×(♂+ wLhet1 + wLhet2 + <u>wLhet3</u>)

on the one hand and

- type [b]: (♀+ wLhet1)×(♂+ wLhet1 + <u>wLhet2</u>) and (♀+ wLhet1 + wLhet3)×(♂+ wLhet1 + <u>wLhet2</u> + wLhet3)

on the other hand (the *Wolbachia* strain that is exclusively harbored by males is underscored because it is this strain that tips the scale). In contrast, differences in CI level *between* any combination of crosses of type [a] and type [b] are not forbidden. ANOVA tests showed indeed that there is no significant difference in CI levels within the equivalent crosses of type [a] or within the equivalent crosses of type [b]. Nevertheless, CI levels differed in any combination of crosses [a] and [b]. This is exactly what the goalkeeper-model would predict if in incompatible crosses, CI levels are determined by the norm approach to CI levels.

Effects of multiple infections have also been studied in the flower bug *Orius strigicollis* [6]. The authors' results showed that, for the two cases that were statistically significant, CI levels in males infected with two *Wolbachia* strains were not higher than in single infections. While this result stands in contrast to our predictions of type 2, the authors provide a likely explanation for this unexpected finding. When they tested for *Wolbachia* density in the host, they found reduced densities in hosts with multiple infections. Decreased density probably leads to decreased production of mod factors and thus to lower CI levels. As a result, even though other empirical studies did not find a similar effect of multiple infections on *Wolbachia* density [4, 5], one may have to correct for possible density reductions when making tests like those presented in this section. Conversely, other aspects of the work on *Orius* [6] are in support of our predictions. Specifically, two statistically significant examples where more *Wolbachia* strains in females decrease CI levels are in accord with our predictions of type 1. Moreover, the fact that eight of ten possible comparisons of equivalent crosses did not show significant differences is in line with our predictions of type 3. We caution, however, that trying *not* to find significant differences may lead to false positive results.

References

1. Zabalou S, Apostolaki A, Pattas S, Veneti Z, Paraskevopoulos C, et al. (2008) Multiple rescue factors within a Wolbachia strain. Genetics 178: 2145–2160.

2. Sinkins SP, W T, Lynd AR, Steven AR, Makepeace BL, et al. (2005) Wolbachia variability and host effects on crossing type in culex mosquitoes. Nature 436: 257–260.

3. Engelstädter J, Hammerstein P, Hurst GDD (2007) The evolution of endosymbiont density in doubly infected host species. Journal of Evolutionary Biology 20: 685–695.

4. Mouton L, Henri H, Bouletreau M, Vavre F (2003) Strain-specific regulation of intracellular Wolbachia density in multiply infected insects. Molecular ecology 12: 3459–3465.

5. Mouton L, Dedeine F, Henri H, Boulétreau M, Profizi N, et al. (2004) Virulence, multiple infections and regulation of symbiotic population in the Wolbachia-Asobara tabida symbiosis. Genetics 168: 181.

6. Watanabe M, Miura K, Hunter M, Wajnberg E (2010) Superinfection of cytoplasmic incompatibility-inducing Wolbachia is not additive in *Orius strigicollis* (Hemiptera: Anthocoridae). Heredity : 1–7.

7. Callaini G, Dallai R, Riparbelli MG (1997) Wolbachia-induced delay of paternal chromatin condensation does not prevent maternal chromosomes from entering anaphase in incompatible crosses of *Drosophila simulans*. J Cell Sci 110 (Pt 2): 271–280.

8. Serbus LR, Casper-Lindley C, Landmann F, Sullivan W (2008) The genetics and cell biology of Wolbachia-host interactions. Annual Review of Genetics 42: 683–707.

9. Poinsot D, Charlat S, Merçot H (2003) On the mechanism of Wolbachia-induced cytoplasmic incompatibility: confronting the models with the facts. Bioessays 25: 259–265.

10. Merçot H, Llorente B, Jacques M, Atlan A, Montchamp-Moreau C (1995) Variability within the seychelles cytoplasmic incompatibility system in *Drosophila simulans*. Genetics 141: 1015–1023.

11. Sinkins SP, Braig HR, O'Neill SL (1995) Wolbachia superinfections and the expression of cytoplasmic incompatibility. Proc R Soc B 261: 325–330.

12. Rousset F, Braig HR, O'Neill SL (1999) A stable triple Wolbachia infection in Drosophila with nearly additive incompatibility effects. Heredity 82: 620–627.

13. Mouton L, Henri H, Boulétreau M, Vavre F (2005) Multiple infections and diversity of cytoplasmic incompatibility in a haplodiploid species. Heredity 94: 187–192.
